# Awake prone positioning does not reduce the risk of intubation in COVID-19 treated with high-flow nasal oxygen therapy: a multicenter, adjusted cohort study

**DOI:** 10.1186/s13054-020-03314-6

**Published:** 2020-10-06

**Authors:** Carlos Ferrando, Ricard Mellado-Artigas, Alfredo Gea, Egoitz Arruti, César Aldecoa, Ramón Adalia, Fernando Ramasco, Pablo Monedero, Emilio Maseda, Gonzalo Tamayo, María L. Hernández-Sanz, Jordi Mercadal, Ascensión Martín-Grande, Robert M. Kacmarek, Jesús Villar, Fernando Suárez-Sipmann, for the COVID-19 Spanish ICU Network, Marina Vendrell, Gerard Sánchez-Etayo, Amalia Alcón, Isabel Belda, Mercé Agustí, Albert Carramiñana, Isabel Gracia, Miriam Panzeri, Irene León, Jaume Balust, Ricard Navarro, María José Arguís, María José Carretero, Cristina Ibáñez, Juan Perdomo, Antonio López, Manuel López-Baamonde, Tomás Cuñat, Marta Ubré, Antonio Ojeda, Andrea Calvo, Eva Rivas, Paola Hurtado, Roger Pujol, Nuria Martín, Javier Tercero, Pepe Sanahuja, Marta Magaldi, Miquel Coca, Elena del Rio, Julia Martínez-Ocon, Paula Masgoret, Angel Caballero, Raquel Risco, Lidia Gómez, Nicolás de Riva, Ana Ruiz, Beatriz Tena, Monserrat Tio, Sebastián Jaramillo, José María Balibrea, Francisco Borja de Lacy, Ana Otero, Ainitze Ibarzabal, Raquel Bravo, Anna Carreras, Daniel Martín-Barreda, Alfonso Jesús Alias, Mariano Balaguer, Jorge Aliaga, Alex Almuedo, Joan Ramón Alonso, Rut Andrea, Gerard Sergi Angelès, Marilyn Arias, Fátima Aziz, Joan Ramon Badía, Enric Barbeta, Toni Torres, Guillem Batiste, Pau Benet, Xavi Borrat, María Borrell, Ernest Bragulat, Inmaculada Carmona, Manuel Castellà, Pedro Castro, Joan Ceravalls, Oscar Comino, Claudia Cucciniello, Clàudia De Deray, Oriol De Diego, Paula De la Matta, Marta Farrero, Javier Fernández, Sara Fernández, Anna Fernández, Miquel Ferrer, Ana Fervienza, María Tallo Forga, Daniel Forné, Clàudia Galán, Andrea Gómez, Eduard Guasch, María Hernández- Tejero, Adriana Jacas, Beltrán Jiménez, Pere Leyes, Teresa López, José Antonio Martínez, Graciela Martínez-Pallí, Jordi Mercadal, Guido Muñoz, José Muñoz, Josep María Nicolás, José Tomás Ortiz, Anna Peiró, Manuel Pérez, Esteban Poch, Margarida Pujol, Eduard Quintana, Bartomeu Ramis, Enric Reverter, Irene Rovira, Pablo Ruiz, Elena Sandoval, Stefan Schneider, Oriol Sibila, Carla Solé, Alex Soriano, Dolors Soy, M. Suárez, Adrián Téllez, Néstor David Toapanta, Antoni Torres, Xavier Urra, César Aldecoa, Alicia Bordell, Silvia Martín, Judith Andrés, Alberto Martínez Ruiz, Gonzalo Tamayo Medel, Iñaki Bilbao Villasante, Fernando Iturri Clavero, Covadonga Peralta Álvarez, Julia T. Herrera Díez, Andrea García Trancho, Iñaki Sainz Mandiola, Carmen Ruano Suarez, Angela Ruiz Bocos, Eneritz Urrutia Izagirre, Pablo Ortiz de Urbina Fernández, Naiara Apodaka López, Leire Prieto Molano, Eunate Ganuza Martínez, Iratxe Vallinas Hidalgo, Karmele de Orte Sancho, Celia González Paniagua, Gemma Ortiz Labrador, Mireia Pérez Larrañaga, Marta López Miguelez, Estíbaliz Bárcena Andrés, Erik Urutxurtu Laureano, Maria Jesús Maroño Boedo, Blanca Escontrela Rodríguez, Aitziber Ereñozaga Camiruaga, Deiene Lasuen Aguirre, Ainhoa Zabal Maeztu, Ane Guereca Gala, Iker Castelo Korro, Andrés Álvarez Campo, Alejandro Carcelen Viana, Alejandro Alberdi Enríquez, Xabier Ormazábal Rementeria, Alberto Sánchez Campos, Rosa Gutiérrez Rico, Pablo Barbier Damborenea, Marta Guerenabarrena Momeñe, Borja Cuesta Ruiz, Alejandro López Rico, Ana Rojo Polo, Covadonga García Grijelmo, Mikel Celorrio Reta, Eneko Martín Arroyo, Leire Artaza Aparicio, Iñaki Ituarte Aspiazu, Ane Igeregi Basabe, Itxaso Merino Julian, Isabel Diaz Rico, Maria Paz Martínez, Ramón Adalia, Luigi Zattera, Irina Adalid Hernández, Leire Larrañaga Altuna, Aina Serrallonga Castells, Adriana Vilchez Garcia, María Núñez, Lorena Román, Francisco Javier Redondo Calvo, Rubén Villazala González, Victor Baladron González, Patricia Faba, Omar Montenegro, Natalia Bejarano Ramírez, Sergio Marcos Contreras, Alejandro Garcia Rodríguez, Saleta Rey Vázquez, Cristina Garcia Pérez, Eva Higuera Miguelez, Irene Pérez Blanco, David García Rivera, Ane Martín de la Fuente, Marta Pardo, Vanessa Rodriguez, Unai Bengoetxea, Fernando Ramasco, Sheila Olga Santidrián Bernal, Alvar Santa Cruz Hernando, Antonio Planas Roca, Carlos Figueroa Yusta, Esther García Villabona, Carmen Vallejo Lantero, Eva Patiño Rodriguez, Alvaro Esquivel Toledo, David Arribas Méndez, Mar Orts Rodriguez, Rosa Méndez Hernández, Jesús Nieves Alonso, Inés Imaz Artazcoz, Sonia Expósito Carazo, Carlos Román Guerrero, Elena Rojo Rodríguez, Ricardo Moreno González, Julia Hernando Santos, Jara Torrente Pérez, Esperanza Mata Mena, Manuel José Muñoz Martínez, Enrique Alday Muñoz, Patricia Martin Serrano, Laura Cotter Muñoz, Amadea Mjertan, Diego Gutierrez Martínez, Carmen Rodríguez García, Olaya Alonso Viejo, Juan Alvarez Pereira, Ana Carmona Bonet, Diana Parrado López, Eva de Dios Tomas, Rafael Martín Celemin, María Luisa Meilan Paz, Luis Quecedo Gutiérrez, Noemí Diaz Velasco, Gabriel Martin Hernández, Francisco Garcia del Corral, Gloria Hernandez Arias, David Rodriguez Cuesta, Ana Gómez Rice, Encarna Mateos Sevillano, Natalia Olmos Molpeceres, Beatriz Domínguez, Ana Vázquez Lima, Ángel Candela, Ismael A. Acevedo Bambaren, Maria Isabel Albala Blanco, Paloma Alonso Montoiro, Fernando Álvarez Utrera, Juan Avellanosa Esteruelas, Amal Azzam López, Alberto José Balvis Balvis, Tommaso Bardi, María Beltrán Martín, Jacobo Benatar Haserfaty, Alberto Berruezo Camacho, Laura Betolaza Weimer, María del Mar Carbonell Soto, Cristina Carrasco Seral, Cristina Cerro Zaballos, Elizabeth Claros Llamas, Pilar Coleta Orduna, Ingrid P. Cortes Forero, Pascual Agustín Crespo Aliseda, María Angélica de Pablo Pajares, Yolanda Díez Remesal, Trinidad Dorado Díaz, Noemí Echevarría Blasco, María Elena Elías Martín, Javier Felices Triviño, Natalia Fernández López, Cristina Fernández Martín, Natalia Ferreiro Pozuelo, Luis Gajate Martín, Clara Gallego Santos, Diego Gil Mayo, María Gómez Rojo, Claudia González Cibrián, Elena Herrera López, Borja Hinojal Olmedillo, Berta Iglesias Gallego, Sassan Khonsari, María Nuria Mane Ruiz, María Manzanero Arroyo, Ana María Mariscal Ortega, Sara Martín Burcio, María del Carmen Martín González, Ascensión Martín Grande, Jose Juan Martín López, Cecilia Martín Rabes, Marcos Martínez Borja, Nilda Martínez Castro, Adolfo Martínez Pérez, Snejana Matcan, Cristina Medrano Viñas, Lisset Miguel Herrera, Adrián Mira Betancur, María Montiel Carbajo, Javier Moya Moradas, Lorena Muñoz Pérez, Mónica Nuñez Murias, Eva Ordiales González, Óscar Ordoñez Recio, Miguel Ángel Palomero Rodriguez, Diego Parise Roux, Lucia Pereira Torres, David Pestaña Lagunas, Juana María Pinto Corraliza, Marian Prieto Rodrigo, Inmaculada Rodriguez Diaz-Regaño, David Rodriguez Esteban, Víctor Rojas Pernia, Álvaro Ruigómez Saiz, Bárbara Saavedra Villarino, Noemí Samaranch Palero, Gloria Santos Pérez, Jaume Serna Pérez, Ana Belén Serrano Romero, Jesús Tercero López, Carlos Tiscar García, Marta de la Torre Concostrina, Eva María Ureta Mesa, Eva Velasco Olarte, Judith Villahoz Martínez, Raúl Villalaba Palacios, Gema Villanueva García, Cristina Vogel de Medeiros, Soraya Gholamian Ovejero, Marta Vicente Orgaz, Patricia Lloreda Herradon, Cristina Crespo Gómez, Tatiana Sarmiento-Trujillo, Noemí García Medina, María Martínez García, Carles Espinós Ramírez, Nabil Mouhaffel Rivero, Jose Antonio Bernia Gil, Sonsoles Martín, María Victoria Moral, Josefina Galán, Pilar Paniagua, Sergio Pérez, Albert Bainac, Ana Arias, Elsa Ramil, Jorge Escudero, Pablo Monedero, Carmen Cara, Andrea Lara, Elena Mendez Martínez, Jorge Mendoza, Íñigo Rubio Baines, Carmen Sala Trull, Pablo Montero López, Alfredo Gea, Alejandro Montero, Rocío Armero Ibañez, Juan Vicente Llau Pitarch, Fernando Rauer Alcóver, Cristina Álvarez Herreros, Cyntia Sánchez Martín, Lucía López Ocáriz Olmos, Marta Navas Moruno, Fernando García Montoto, Mirón Rodriguez, Laura Fuentes Coco, Cristina Hernández Gamito, Antonio Barba Orejudo, Luis Gerardo Smith Vielma, Yasmina González Marín, Francisco de Borja Amador Penco, Marta Donoso Domínguez, Silvia Esquivel Ramírez, José Antonio Carbonell, Berta Monleón López, Sara Martínez-Castro, Gerardo Aguilar, María Gestal, Pablo Casas, Angel Outeiro Rosato, Andrea Naveiro Pan, María Alonso Portela, Adrián García Romar, Eva Mosquera Rodríguez, Diego Ruanova Seijo, Pablo Rama Maceiras, Francisco Castro-Ceoane, Esther Moreno López, Sergio Gil, Julia Guillén Antón, Patricia García-Consuegra Tirado, Aurora Callau Calvo, Laura Forés Lisbona, María Carbonell Romero, Belén Albericio Gil, Laura Pradal Jarne, María Soria Lozano, Diego Loscos López, Andrea Patiño Abarca, Jordi Serrano, Javier Pérez-Asenjo, Ángel Díez-Domínguez, Ion Zubizarreta, Jon Ramos, Iosu Fernández, Emilio Maseda, Alejandro Suárez de la Rica, Javier Veganzones, Itziar Insausti, Javier Sagra, Sofía Díaz Carrasco, Ana Montero Feijoo, Julio Yagüe, Ignacio Garutti, Eva Bassas Parga, Carmen Deiros Garcia, Elisenda Pujol Rosa, Ana Tejedor Navarro, Roser Font Gabernet, Maria José Bernat, Meritxell Serra Valls, Cristina Cobaleda Garcia-Bernalt, Jesus Fernanz Anton, Adriana Aponte Sierra, Lucia Gil Gomez, Olaia Guenaga Vaqueiro, Susana Hernandez Marin, Laura Pardo Pinzon, Sira Garcia Aranda, Carlos Briones Orejuela, Edgar Cortes Sanchez, Alejandro Romero Fernandez, Esther Fernández Sanjosé, Patricia Iglesias Garsabal, Guillermo Isidro Lopez, Ana Vicol, Sara Espejo Malagon, María Sanabra Loewe, Laura Grau Torradeflo, Lourdes Blanco Alcaide, Gloria Buenaventura Sanclemente, Pere Serra Pujol, Gustavo Cuadros Mendoza, Miroslawa Konarska, Fedra Bachs Almenara, Agnieszka Golska, Aleix Carmona Blesa, Arantxa Mas Serra, Javier Ripolles Melchor, Ana Nieto Moreno, Káteri Chao Novo, Sandra Gadín López, Elena Nieto Moreno, Bérénice Gutiérrez Tonal, Elena Lucena de Pablo, Barbara Algar Yañez, Beatriz Vázquez Rivero, Beatriz Nozal Mateo, Marina de Retes, Norma Aracil Escoda, Cristina Gallardo Mayo, Rosa Sanz González, Alicia Ruiz Escobar, Maria Laura Pelegrina López, Marina Valenzuela Peña, David Stolle Dueñas, Ane Abad Motos, Alfredo Abad-Gurumeta, Ana Tirado Errazquin, Elena Sáez Ruiz, Nerea Gómez Pérez, Francisco de Borja Bau González, Cesar Morcillo Serra, Jessica Souto Higueras, Rosario Vicente, Raquel Ferrandis, Silvia Polo Martín, Azucena Pajares Moncho, Ignacio Moreno Puigdollers, Juan Pérez Artacho Cortés, Ana Moret Calvo, Ana Pi Peña, María Catalán Fernández, Marina Varela, Pilar Díaz Parada, Raquel Rey Carlín, Sarra Barreiro Aragunde, María Isabel Forés Chiva, A. Javier Agulló, Antonio Pérez Ferrer, María Galiana, Antoni Margarit, Válerie Mourre del Rio, Eva Heras Muxella, Anna Vidal

**Affiliations:** 1 Department of Anesthesiology and Critical Care, Hospital Clínic, Institut D’investigació August Pi i Sunyer, Villarroel 170, 08036 Barcelona, Spain; 2 CIBER de Enfermedades Respiratorias, Instituto de Salud Carlos III, Madrid, Spain; 3 Department of Preventive Medicine and Public Health, University of Navarra, Pamplona, Spain; 4 Ubikare Technology, Vizcaya, Spain; 5 Department of Anesthesiology and Critical Care, Hospital Universitario Río Hortega, Valladolid, Spain; 6 Department of Anesthesiology and Critical Care, Hospital del Mar, Barcelona, Spain; 7 Department of Anesthesiology and Critical Care, Hospital Universitario La Princesa, Madrid, Spain; 8 Department of Anesthesiology and Intensive Care, Clínica Universitaria de Navarra, Pamplona, Spain; 9 Department of Anesthesiology and Critical Care, Hospital Universitario La Paz, Madrid, Spain; 10 Department of Anesthesiology and Critical Care, Hospital Universitario de Cruces, Barakaldo, Vizcaya Spain; 11 Department of Anesthesiology and Critical Care, Hospital Universitario Ramón y Cajal, Madrid, Spain; 12 Department of Respiratory Care, Massachusetts General Hospital, Boston, MA USA; 13 Multidisciplinary Organ Dysfunction Evaluation Research Network, Research Unit, Hospital Universitario Dr. Negrin, Las Palmas de Gran Canaria, Spain; 14 Intensive Care Unit, Hospital Universitario La Princesa, Madrid, Spain

**Keywords:** Acute respiratory failure, COVID-19, High-flow nasal oxygen therapy, Prone positioning, Mechanical ventilation, Critical care

## Abstract

**Background:**

Awake prone positioning (awake-PP) in non-intubated coronavirus disease 2019 (COVID-19) patients could avoid endotracheal intubation, reduce the use of critical care resources, and improve survival. We aimed to examine whether the combination of high-flow nasal oxygen therapy (HFNO) with awake-PP prevents the need for intubation when compared to HFNO alone.

**Methods:**

Prospective, multicenter, adjusted observational cohort study in consecutive COVID-19 patients with acute respiratory failure (ARF) receiving respiratory support with HFNO from 12 March to 9 June 2020. Patients were classified as HFNO with or without awake-PP. Logistic models were fitted to predict treatment at baseline using the following variables: age, sex, obesity, non-respiratory Sequential Organ Failure Assessment score, APACHE-II, C-reactive protein, days from symptoms onset to HFNO initiation, respiratory rate, and peripheral oxyhemoglobin saturation. We compared data on demographics, vital signs, laboratory markers, need for invasive mechanical ventilation, days to intubation, ICU length of stay, and ICU mortality between HFNO patients with and without awake-PP.

**Results:**

A total of 1076 patients with COVID-19 ARF were admitted, of which 199 patients received HFNO and were analyzed. Fifty-five (27.6%) were pronated during HFNO; 60 (41%) and 22 (40%) patients from the HFNO and HFNO + awake-PP groups were intubated. The use of awake-PP as an adjunctive therapy to HFNO did not reduce the risk of intubation [RR 0.87 (95% CI 0.53–1.43), *p* = 0.60]. Patients treated with HFNO + awake-PP showed a trend for delay in intubation compared to HFNO alone [median 1 (interquartile range, IQR 1.0–2.5) vs 2 IQR 1.0–3.0] days (*p* = 0.055), but awake-PP did not affect 28-day mortality [RR 1.04 (95% CI 0.40–2.72), *p* = 0.92].

**Conclusion:**

In patients with COVID-19 ARF treated with HFNO, the use of awake-PP did not reduce the need for intubation or affect mortality.

## Background

A high number of patients with coronavirus disease 19 (COVID-19) develop severe bilateral viral pneumonia. Many COVID-19 patients evolve to acute respiratory distress syndrome (ARDS), characterized by profound hypoxemia and an associated high mortality rate [1, 2]. High-flow nasal oxygen therapy (HFNO) is effective in decreasing the need for endotracheal intubation in patients with acute hypoxemic respiratory failure (ARF) [3]. However, the lack of proven benefits in COVID-19 patients together with the concerns of increased risk of aerosolization led to recommending early intubation and invasive mechanical ventilation (MV) at the beginning of the pandemic. Due to the high infection rate of COVID-19, this resulted in a rapid exhaustion of ICU resources worldwide [4].

However, MV is associated with substantial risks including ventilator-associated pneumonia, ICU-acquired weakness, delirium, and cognitive impairment. The recognition that the potential benefits of HFNO for preventing intubation and sparing critical ICU resources could outweigh its risks soon led to guidelines and expert recommendations advocating its use during the pandemic [5–7]. Nevertheless, when choosing HFNO to support COVID-19-related ARF, two considerations should be made. First, HFNO may be insufficient to correct the hypoxemia secondary to intrapulmonary shunt and ventilation-perfusion (V/Q) mismatch. Second, it may delay intubation and invasive MV, which may worsen the patients’ outcome, as suggested in ARDS patients [8]. Vigorous breathing efforts in hypoxemic ARF patients promoting further lung injury (a process known as patient self-inflicted lung injury, P-SILI) may worsen the outcome [9]. In this context, the use of awake prone positioning (awake-PP) during spontaneous breathing in non-intubated patients could contribute to a reduction of the risk of P-SILI by promoting a more homogeneous distribution of ventilation while improving oxygenation and V/Q matching [10].

Several studies have shown that the combination of awake-PP and HFNO or non-invasive ventilation (NIV) is feasible in patients with severe COVID-19 pneumonia, resulting in an increase in oxygenation or a decrease in the respiratory rate and/or dyspnea [11–16]. However, to date, it has not been established whether the combination of HFNO plus awake-PP could prevent the need for invasive MV and decrease the need of ICU resources in COVID-19 patients with ARF. We performed this large multicenter adjusted cohort study to investigate those issues.

## Material and methods

### Study design

This is a prospective, multicenter, adjusted cohort study of consecutive patients with COVID-19 ARDS admitted to 36 hospitals from Spain and Andorra. The study was approved (additional file 2) by the referral Ethics Committee (Hospital de Cruces, Vizcaya, Spain) and by all participating centers. Each participating center considered the need for written informed consent. This study followed the “Strengthening the Reporting of Observational Studies in Epidemiology (STROBE)” guidelines for observational cohort studies [17].

### Study population and data collection

Data from patients’ electronic medical records were reviewed and collected by physicians trained in critical care according to a previously standardized common protocol. Each investigator had a personal username/password and entered data into a specifically pre-designed online data acquisition system (CoVid19.ubikare.io) endorsed and validated by the Spanish Society of Anesthesiology and Critical Care (SEDAR) (https://www.sedar.es/images/site/REGISTRO_CRITICOS_COVID19/MANUAL_REGISTRO_REG-SARS-COVID19.pdf). Patient confidentiality was protected by assigning a de-identified patient code. All consecutive COVID-19 patients included in the dataset from March 12th to June 9th, 2020, were enrolled if they fulfilled the following criteria: (1) age ≥ 18 years, (2) confirmed SARS-CoV-2 infection from a respiratory tract sample using PCR-based tests, (3) no previous invasive MV or NIV use before starting HFNO, and (4) peripheral oxyhemoglobin saturation (SpO_2_) < 93% with a non-rebreather face mask at 15 L/min. Patients with non-confirmed SARS-CoV-2 infection according to WHO guidance and patients with no data on ventilation strategies were excluded.

Recorded data included demographics [age, gender, body mass index (BMI)], comorbidities, previous pharmacological treatments, disease chronology [time from onset of symptoms and from hospital admission to initiation of respiratory support, ICU length of stay (LOS)], symptoms at ICU admission, vital signs [temperature, mean arterial pressure (MAP), heart rate], laboratory parameters (blood test, coagulation, biochemical), non-respiratory Sequential Organ Failure Assessment (non-respiratory SOFA) and APACHE II scores, patients requiring invasive MV, patients discharged from ICU, and patients who had died or were still under ICU care on June 28, 2020.

We defined baseline as the first day on HFNO and collected a full set of data on that day. Site investigators collected what they considered the representative data of each day from admission to ICU discharge. We also collected the “worst” values during the study period (maximum or minimum, depending on the variable). In the case report form, prone position was only considered if the duration was > 16 h/day regardless of the number of sessions. Before data were analyzed, two independent investigators and a statistician screened for erroneous data against standardized ranges and contacted local investigators with any queries. Only validated or corrected data were entered into the database. For the purpose of this analysis, patients were classified into two groups: (1) patients who received HFNO + awake-PP and (2) patients who only received HFNO. Awake-PP was indicated by medical criteria and was not uniformly defined and protocolized for the study.

### Statistical analysis

As this is an observational study, and no harm is inflicted and no benefit associated with being in the study we aimed to recruit as many patients as possible, with no pre-defined sample size. Descriptive variables are expressed as percentage, mean and standard deviation (SD), or median and interquartile range (IQR), as appropriate for each variable. We used the Student *t* test or Mann-Whitney test for numerical variables and chi-squared test or Fisher exact test for categorical variables, to compare variables across groups. We used inverse probability of treatment weighting to account for baseline differences between HFNO and HFNO + awake-PP groups. Based on the literature, we fitted logistic models to predict treatment at baseline using the following variables as predictors of treatment: age, sex, obesity, non-respiratory SOFA score, APACHE II, C-reactive protein (CRP), days from symptoms onset to HFNO initiation, respiratory rate, SpO_2_, and type of hospital (4 groups depending on the number of enrolled patients). Weights were calculated following the methodology described elsewhere and a weighted population (adjusted sample) was built subsequently [18]. To assess the relationship among the exposure awake-PP and the probability of being intubated and mortality at day 28, time to event curves were plotted using the Kaplan-Meier method and analyzed with log-rank test and multivariate Cox regression analysis. For Kaplan-Meier analyses, patients with complementary outcome were right-censored at the longest recorded length of stay. We also stratified patients by PaO_2_/FiO_2_ below or above 100. Missing data were not imputed. Analyses were performed on a complete case analysis basis. All tests were two-sided, and a *P* value < 0.05 was considered statistically significant. All analyses were performed with STATA version 16.

## Results

**Fig. 1:**
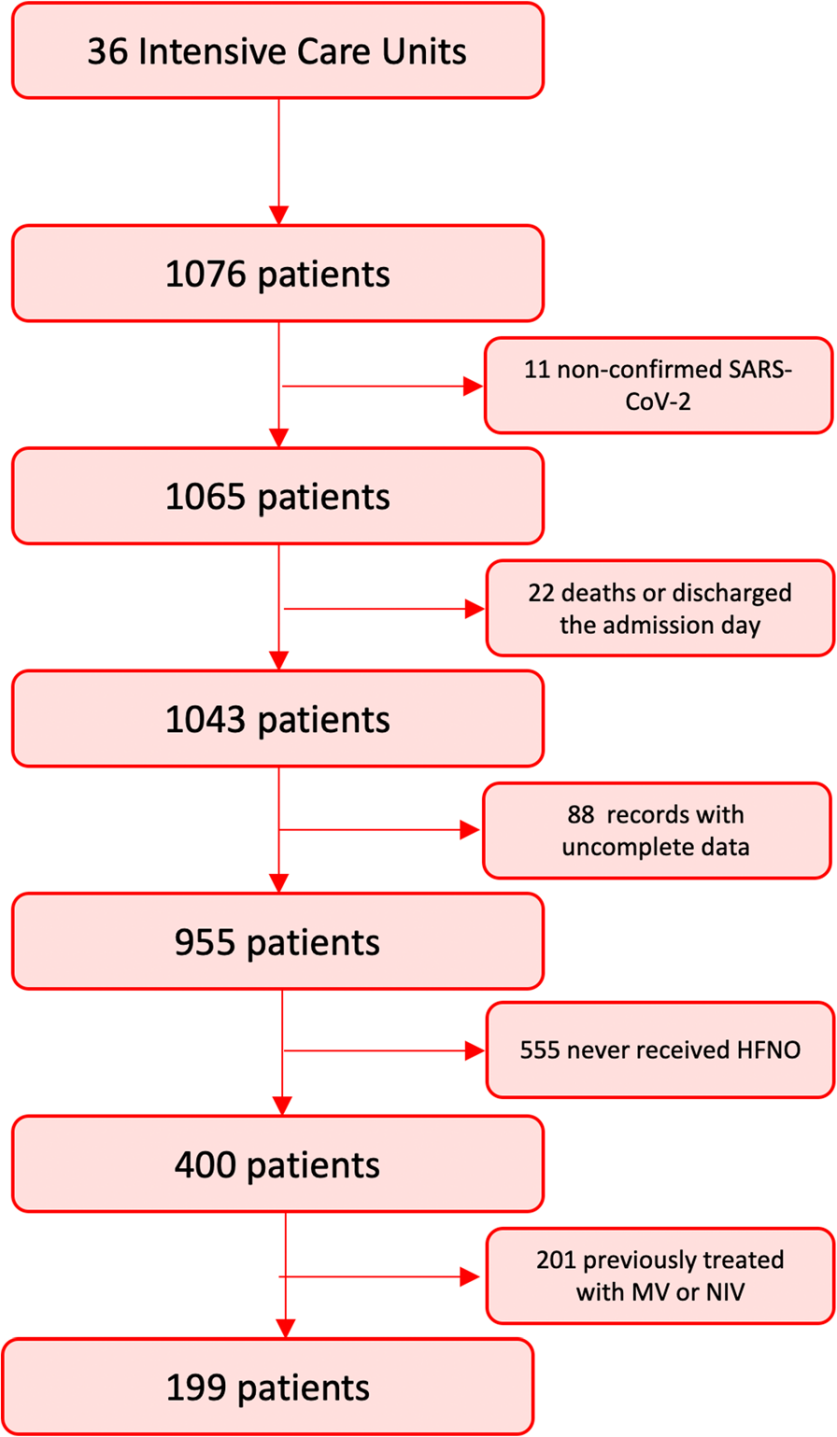
Patient flowchart. HFNO, high-flow nasal oxygen therapy; MV, invasive mechanical ventilation; NIV, noninvasive ventilation

Between March 12th and June 9th, 2020, 1076 critically ill patients admitted in 36 ICUs in Spain and Andorra were included in the database. HFNO was used in 400 patients during their ICU stay, but in 199 patients, HFNO was the first therapeutic option (Fig. 1). From those 199 patients, 55 (27.6%) were pronated during HFNO. The median time from symptom onset to hospital admission and to HFNO or HFNO + awake-PP start were 7 vs 7 days and 10 vs 11 days, respectively (Table 1).

**Table 1:** Baseline characteristics of the original-eligible population and weighted population

	Original sample			Weighted sample		
	HFNO (*n* = 144)	HFNO + awake-PP (*n* = 55)	*P* value	HFNO 68.43%	HFNO + awake-PP 31.57%	*P* value
**Patients demographics and comorbidities**						
Age	63.0 [55.0–71.0]/144	60.0 [54.0–70.0]/55	0.38	60.3	60.9	0.82
Gender, female	39/143 (27.3%)	13/54 (24.1%)	0.71	28.8%	33.9%	0.62
Body mass index, kg/m^2^	27.3 [25.1–29.4]/120	26.8 [24.8–31.2]/49	0.75	28.6	28.2	0.66
Arterial Hypertension	60/144 (41.7%)	20/55 (36.4%)	0.52	42.8%	34.3%	0.41
Diabetes Mellitus	23/144 (16.0%)	9/55 (16.4%)	0.99	18.1%	10.7%	0.25
Chronic heart failure	2/144 (1.4%)	2/55 (3.6%)	0.30	1.4%	5.2%	0.46
Chronic renal failure	14/144 (9.7%)	4/55 (7.3%)	0.78	6.4%	6.2%	0.98
Asthma	5/144 (3.5%)	1/55 (1.8%)	0.99	7.6%	6.3%	0.87
COPD	6/144 (4.2%)	4/55 (7.3%)	0.46	4.2%	8.2%	0.44
Obesity	25/120 (20.8%)	17/49 (34.7%)	0.07	30.2%	32.4%	0.82
Dyslipidemia	15/144 (10.4%)	4/55 (7.3%)	0.59	8.1%	4.5%	0.38
Malignancy	9/144 (6.3%)	3/55 (5.5%)	0.99	4.9%	3.2%	0.68
**Medical treatment**						
Anti-hypertensive agents	62/144 (43.1%)	19/55 (34.6%)	0.33	43.9%	35.9%	0.45
Hypoglycemic agents	18/144 (12.5%)	7/55 (12.7%)	0.99	17.8%	17.0%	0.92
Antiplatelet agents	17/144 (11.8%)	5/55 (9.1%)	0.80	8.8%	12.8%	0.55
Anticoagulants	10/144 (6.9%)	1/55 (1.8%)	0.29	10.7%	1.2%	0.014
Bronchodilators	35/144 (24.3%)	10/55 (18.2%)	0.44	22.4%	23.3%	0.93
Lipid lowering agents	8/144 (5.6%)	3/55 (5.5%)	0.99	7.8%	3.2%	0.32
Thyroid hormone replacement	10/144 (6.9%)	9/55 (16.4%)	0.058	12.4%	25.5%	0.20
Immunossupressors	9/144 (6.3%)	1/55 (1.8%)	0.29	4.1%	0%	0.050
Corticosteroids	9/144 (6.3%)	2/55 (3.6%)	0.73	4.1%	0%	0.050
**Chronology**						
Days from symptom onset to hospital admission	7.0 [4.0–9.0]/141	7.0 [4.0010.0]/55	0.75	7.4	7.6	0.79
Days from symptom onset to HFNO	10.0 [8.0–13.0]/142	11.0 [8.0–13.0]/55	0.44	10.1	10.2	0.99
**Symptoms at ICU admission**						
Fever	121/144 (84.0%)	51/55 (92.7%)	0.16	87.0%	90.0%	0.70
Cough	94/144 (65.3%)	36/55 (65.5%)	0.99	69.3%	62.2%	0.50
Dyspnea	92/144 (63.9%)	39/55 (70.9%)	0.40	62.4%	73.8%	0.23
Malaise	57/144 (39.6%)	27/55 (49.1%)	0.26	42.1%	56.3%	0.19
Myalgia	22/144 (15.3%)	10/55 (18.2%)	0.66	18.0%	18.8%	0.92
Headache	12/144 (8.3%)	6/55 (10.9%)	0.58	7.8%	5.8%	0.64
Rhinorrhea	1/144 (0.7%)	1/55 (1.8%)	0.47	1.1%	3.3%	0.52
Vomiting	10/144 (6.9%)	4/55 (7.3%)	0.99	4.6%	7.9%	0.56
Arthralgia	6/144 (4.2%)	4/55 (7.3%)	0.46	3.4%	5.5%	0.63
Chest pain	12/144 (8.3%)	1/55 (1.8%)	0.11	9.2%	0%	0.006
Increased sputum	14/144 (9.7%)	6/55 (10.9%)	0.79	7.7%	11.0%	0.57
Anosmia	6/144 (4.2%)	4/55 (7.3%)	0.46	6.5%	6.5%	0.99
Pharyngodynia	5/144 (3.5%)	1/55 (1.8%)	0.99	3.5%	1.2%	0.33
Diarrhea	20/144 (13. 9%)	9/55 (16.4%)	0.65	15.8%	15.0%	0.91
Fatigue	1/144 (0.7%)	4/55 (7.3%)	0.021	0%	6.6%	0.052
**Scores**						
APACHE II	11.0 [8.0–14.0]/107	8.5 [6.0–13.0]/46	0.069	10.8	11.0	0.87
Non-respiratory SOFA	4.0 [4.0–5.0]/116	4.0 [4.0–4.0]/46	0.11	4.6	4.7	0.93
**Vital Signs**						
Temperature, °C	36.9 [36.1–37.6]/141	36.8 [36.2–37.3]/54	0.79	36.9	36.8	0.82
Mean arterial pressure, mmHg	87.3 [79.7–95.0]/142	85.8 [78.0–92.0]/54	0.10	89.1	82.9	0.006
Heart rate, bpm	81.0 [73.0–91.0]/141	78.5 [66.0–88.0]/54	0.073	82.5	78.9	0.25
SpO_2_, %	90.0 [88.0–94.0]/141	90.0 [88.0–92.0]/54	0.57	90.4	90.4	0.99
Respiratory rate, bpm	25.0 [22.0–30.0]/136	23.0 [20.0–30.0]/54	0.081	25.7	25.5	0.87
**Arterial blood gas**						
PaO_2_/FiO_2_	111.0 [83.0–144.0]/124	125.0 [99.0–187.0]/51	0.037	123.9	148.2	0.12
PaCO_2_, mmHg	33.1 [30.0–37.0]/129	34.7 [30.8–39.0]/51	0.23	34.7	34.0	0.54
**Laboratory findings**						
Ferritin, ng/mL	1265 [755–1904]/87	934 [597–2092]/41	0.54	1640	1766	0.77
D-Dimer, ng/mL	925 [600.0–1800]/114	931 [549–1790]/48	0.77	1605	1608	0.99
CRP, mg/dL	16.82 [8.31–30.40]/131	21.51 [8.46–145.00]/53	0.20	56.39	57.7	0.93
Lymphocyte count, 10e3/μL	0.61 [0.40–0.90]/132	0.61 [0.40–0.89]/53	0.82	0.8	0.7	0.60
IL-6, pg/mL	135.0 [61.8–202.0]/17	93.0 [35.5–301.0]/11	0.20	186.6	134.4	0.47
LDH, U/L	396.0 [331.0–480.0]/125	380.0 [313.0–528.0]/51	0.27	417.3	434.3	0.61
Leukocytes, 10^3^/μL	7.1 [5.0–11.2]/131	6.5 [4.4–9.0]/52	0.86	8.1	6.7	0.13
Procalcitonin, ng/mL	0.2 [0.1–0.6]/99	0.1 [0.1–0.3]/39	0.17	0.7	0.3	0.071
Platelets, 1000/mm^3^	232.0 [152.0–342.0]/133	233.0 [153.0–274.0]/53	0.12	261.9	221.3	0.043
Bilirrubin, mg/dL	0.6 [0.4–1.0]/124	0. 7 [0.5–0.9]/48	0.51	0.9	0.7	0.12
GPT, U/L	43.5 [23.0–78.0]/130	37.0 [25.5–71.0]/52	0.73	65.5	62.6	0.84
Creatinine, mg/dL	0.8 [0.6–1.1]/132	0.8 [0.7–1.0]/52	0.67	1.0	1.0	0.72
Urea, mg/dL	36.0 [27.2–53.0]/76	33.6 [21.0–49.0]/42	0.39	45.5	33.7	0.019
Troponin, ng/mL	14.0 [4.4–23.4]/69	8.0 [2.8–15.1]/33	0.061	17.3	13.2	0.46
NTproBNP, pg/mL	418.0 [125.5–1529.0]/16	225.5 [50.0–1263.0]/6	0.33	760.1	731.9	0.94
Hematocrit, %	38.0 [35.0–42.0]/126	40.7 [36.0–44.0]/50	0.041	38.7	39.4	0.63
Lactate, mmol/L	1.5 [1.0–2.1]/82	1.6 [1.3–2.00]/33	0.36	1.8	1.8	0.97

Values were obtained from each patient on day 1 of HFNT. Categorical variables are expressed as proportion, and continuous variables as median (IQR) for original-eligible population and percentage and mean for weighted population

*HFNO* high-flow nasal oxygen therapy, *COPD* chronic obstructive pulmonary disease, *SOFA* Sequential Organ Failure Assessment, *CRP* C-reactive protein, *IL* interleukin, *LDH* lactate dehydrogenase, *GPT* glutamate pyruvate transaminase

Patients’ demographics, symptoms at ICU admission, baseline vital signs, arterial blood gases, and laboratory findings according to HFNO or HFNO + awake-PP are shown in Table 1, both in the original and adjusted samples. There were no differences in the time from symptom onset to hospital admission or onset of HFNO (Table 1). No substantial imbalances in patients’ demographics, vital signs, arterial blood gases, and laboratory findings at baseline were observed (Table 1). In both samples, PaO_2_/FiO_2_ was significantly higher in the HFNO + awake-PP group.

**Fig. 2:**
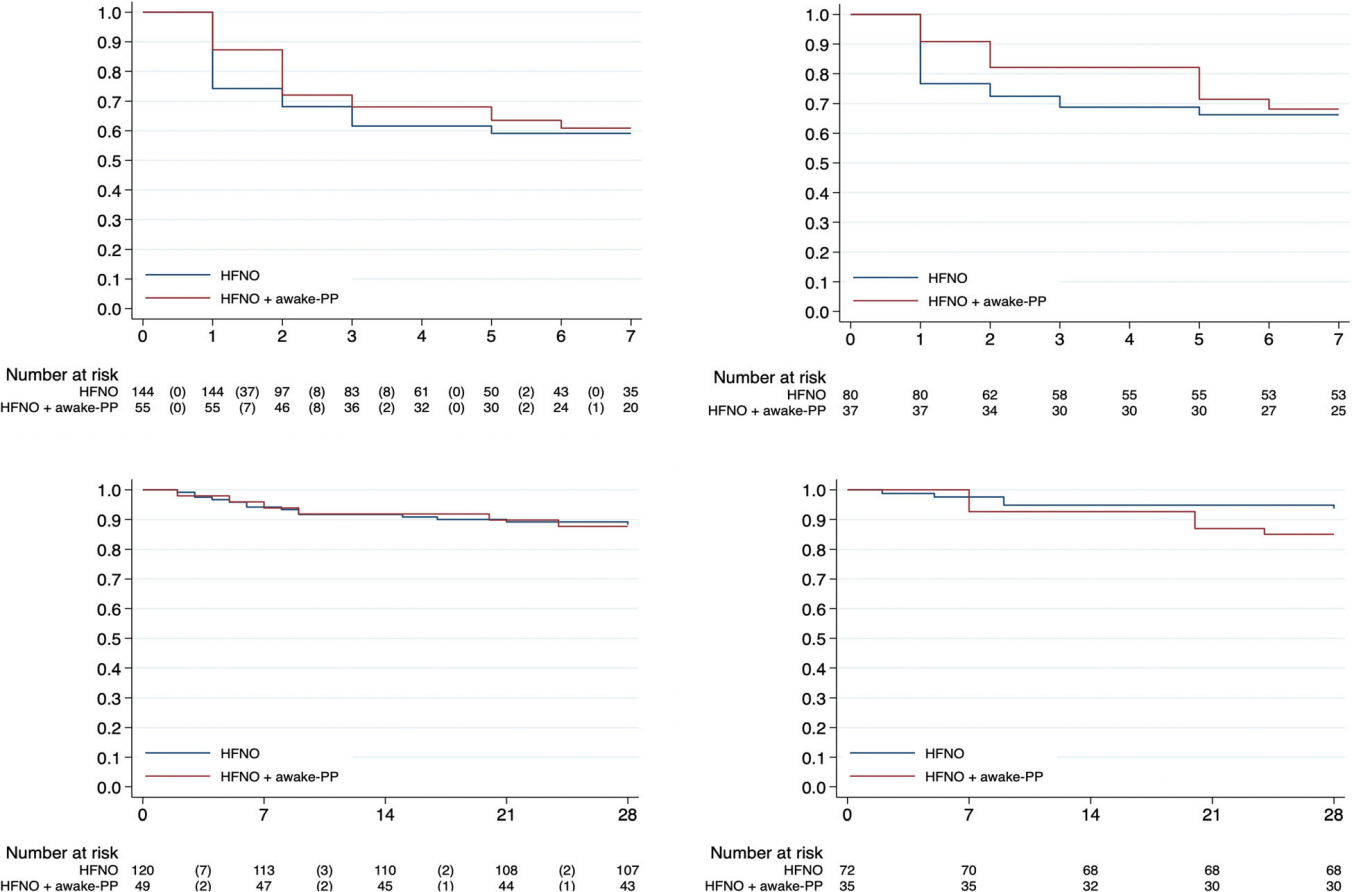
Time to event curves using Kaplan-Meier with multivariate Cox regression. The probability of been intubated in the original (top-left) and weighted (top-right) samples and the probability of 28-day mortality in the original (bottom-left) and weighted (bottom-right) samples were not affected by the use of awake prone positioning. HFNO, high-flow nasal oxygen therapy; HFNO + awake-PP, high-flow nasal oxygen therapy plus awake prone positioning

Table 2 shows the worst patients’ findings during the ICU course while under HFNO treatment in the original and adjusted samples. There were no clinically substantial differences except for IL-6 and procalcitonin levels, both being higher in HFNO patients. Mean values of SpO_2_, RR, and ROX index over time in the adjusted sample are reported in the supplemental digital content 2 (Figures 1, 2 and 3). Differences between the intubated and non-intubated patients in the adjusted sample at baseline and during ICU stay while treated with HFNO are shown in the supplemental digital content 2 (Tables 1, 2, 3 and 4 and Figures 1, 2 and 3).

**Table 2:** Clinical evolution (maximum or minimum values) of the original-eligible population and weighted population while treated with HFNO

°	Original sample			Weighted sample		
	HFNO (*n* = 133)	HFNO + awake-PP (*n* = 51)	*P* value	HFNO 68.4%	HFNO + awake-PP 31.6%	*P* value
**Scores**						
Non-respiratory SOFA	4.0 [4.00–5.00]/125	4.0 [4.00–5.00]/46	0.25	4.8	5.0	0.62
**Vital signs**						
Temperature, °C	37.2 [36.50–38.00]/141	37.1 [36.60–37.80]/54	0.80	37.2	37.3	0.53
Mean arterial pressure, mmHg	77.0 [70.50–83.83]/140	76.2 [68.00–84.00]/54	0.59	77.8	73.4	0.053
Heart rate, bpm	85.0 [75.00–96.00]/141	85.0 [79.00–100.00]/54	0.62	87.2	91.4	0.26
SpO_2_, %	89.0 [86.00–92.00]/141	88.0 [84.00–90.00]/54	0.11	88.8	87.6	0.21
Respiratory rate minimum, bpm	21.0 [18.00–24.00]/141	19.0 [16.00–23.00]/54	0.004	20.8	19.7	0.23
Respiratory rate maximum, bpm	27.0 [24.00–32.00]/141	27.0 [23.00–30.00]/54	0.49	27.7	27.1	0.64
**Arterial blood gas**						
PaO_2_/FiO_2_	92.5 [77.00–125.50]/128	103.0 [80.00–125.00]/53	0.45	109.7	113.8	0.67
PaCO_2_, mmHg	39.9 [35.50–48.00]/131	41.2 [36.20–46.00]/53	0.56	44.8	42.4	0.29
**Laboratory findings**						
Ferritin, ng/mL	1279.0 [694.00–2151.00]/107	1499.0 [809.00–2425.00]/45	0.45	1817.2	1955.0	0.75
D-Dimer, ng/mL	1681.0 [820.00–4200.00]/122	1590.0 [1030.00–3200.00]/50	0.98	2799.7	2624.9	0.76
CRP, mg/dL	21.3 [9.32–33.19]/132	22.7 [8.66–146.14]/53	0.23	62.4	62.6	0.98
Lymphocytes, μL	0.47 [0.30–0.74]/135	0.44 [0.30–0.60]/53	0.31	0.56	0.42	0.021
IL-6, pg/mL	177.0 [42.70–415.90]/17	87.5 [24.00–301.00]/14	0.34	832.7	221.6	0.33
LDH, U/L	429.0 [345.00–561.00]/125	449.0 [352.00–602.00]/51	0.51	451.2	490.3	0.29
Leukocytes, 10^3^/μL	8.3 [5.80–12.00]/122	7.7 [5.21–12.33]/51	0.75	9.7	9.0	0.60
Procalcitonin, ng/mL	0.22 [0.11–0.57]/114	0.20 [0.09–0.34]/45	0.57	1.24	0.34	0.10
Platelets, 1000/mm^3^	319.0 [212.50–410.50]/136	303.0 [244.00–358.00]/53	0.64	330.6	329.7	0.97
Bilirrubin, mg/dL	0.80 [0.50–1.10]/130	0.84 [0.60–1.18]/50	0.33	1.23	0.90	0.052
ALT, U/L	66.0 [30.00–104.00]/135	52.0 [32.00–116.00]/53	0.82	85.2	105.6	0.35
Creatinin, mg/dL	0.90 [0.70–1.18]/136	0.86 [0.75–1.02]/52	0.45	1.10	1.09	0.96
Urea, mg/dL	42.0 [30.00–64.00]/91	39.5 [26.00–61.00]/50	0.44	52.0	42.7	0.12
Troponin, ng/mL	11.8 [4.30–25.00]/89	9.6 [4.60–27.52]/39	0.69	18.8	9.3	0.27
NTproBNP, pg/mL	335.5 [125.50–938.80]/20	303.1 [91.00–1019.00]/14	0.75	727.9	660.9	0.82
Hematocrit, %	38.00 [34.70–42.00]/111	39.20 [36.00–42.50]/45	0.97	38.2	39.4	0.35
Lactate, mmol/L	1.5 [1.16–2.10]/77	1.5 [1.20–2.10]/31	0.60	1.85	1.88	0.91

Maximum or minimum values during the period of HFNO. Categorical variables are expressed as proportion, and continuous variables as median (IQR) for original-eligible population and percentage and mean for weighted population

*HFNO* high-flow nasal oxygen therapy, *SpO*_*2*_ peripheral oxyhemoglobin saturation, *SOFA* Sequential Organ Failure Assessment, *RCP* C-reactive protein, *IL* interleukin, *LDH* lactate dehydrogenase, *GPT* glutamate pyruvate transaminase

From 199 patients, 82 (41%) patients required intubation and invasive MV: 60 (41%) and 22 (40%) in the HFNO and HFNO + awake-PP groups, respectively (Table 5 in the Additional file 1). The use of awake-PP as adjunctive therapy to HFNO did not reduce the risk of being intubated neither in the original nor in the adjusted samples [hazard ratio (RR) 0.87 (95% CI 0.538–1.435), *p* = 0.60] and [RR 1.002 (95% CI 0.531–1.890), *p* = 0.99] (Table 4). HNFO + awake-PP did also not reduce the risk of being intubated in the subgroups of patients with PaO_2_/FiO_2_ greater or less than 100 (Figure 4 in the Additional file 1). Time from HFNO to intubation was longer in the HFNO + awake-PP in the original (1.0 vs 2.0 days, *p* = 0.055) and adjusted (2.0 vs 4.1 days, *p* = 0.054) samples, although differences did not reach statistical significance. As of June 27, 2020, 146 (73%) patients were discharged from the ICU with no differences between HFNO 105 (86%) patients and HFNO + awake-PP 41 (83%) patients (Table 5 in the Additional file 1). ICU length of stay did not vary between groups (7.5 vs 8.0, *p* = 0.27) (Table in the Additional file 1).

The 28-day mortality risk was not influenced by the use of awake-PP [RR 2.411 (95% CI 0.556–10.442), *p* = 0.23)] (Table 3 and Fig. 2). Neither did it influence the subgroups of patients with PaO_2_/FiO_2_ higher or less than 100 (Figure 5 in the Additional file 1).

**Table 3:** Associations between HFNO plus awake prone positioning and the endpoint of intubation and 28-day mortality in the original population and weighted population

Analysis	Hazard ratio (95% CI); *p* value
**Intubation**	
Crude analysis	0.879 (0.538, 1.435); *p* = 0.60
Inverse probability weighting analysis	1.002 (0.531, 1.890); *p* = 0.99
**28-day mortality**	
Crude analysis	1.046 (0.402, 2.722); *p* = 0.92
Inverse probability weighting analysis	2.411 (0.556, 10.442); *p* = 0.23

Logistic models were fitted to predict treatment at baseline using the following variables as predictors of treatment: age, sex, obesity, non-respiratory sequential organ failure assessment severity score, APACHE II, C-reactive protein, days from symptoms onset to high-flow nasal therapy start, respiratory rate, and peripheral oxyhemoglobin saturation

*CI* confidence interval

## Discussion

In this prospective multicenter adjusted study in 199 patients with COVID-19 ARF treated with HFNO, the synergistic use of awake-PP did not reduce the intubation rate. Although 28-day mortality was not affected, our findings also suggest that awake-PP could have a potentially negative impact as it was associated with a delay in intubation. Our analysis does not support widespread use of awake-PP in COVID-19 patients with ARF treated with HFNO. However, given the observational nature of our study, these results should be interpreted with caution and by no means considered definitive.

Published studies on the management of ARF in COVID-19 patients have shown that the vast majority need invasive MV with prolonged times on the ventilator [19, 20]. Alternatives to invasive respiratory support such as HFNO, a simple technique with few side effects, have been widely used during the pandemic. Other adjunctive techniques, such as awake-PP, have been widely used to correct hypoxemia and avoid the need for invasive MV [11–16]. The benefits of prone positioning in ARDS patients have been well established. Prone positioning favors lung recruitment improving V/Q mismatch by decreasing shunt [21, 22]. The resulting more homogeneous distribution of ventilation could decrease the risk of ventilator-induced lung injury, a mechanism directly related to the mortality [23]. However, the experience with awake-PP in ARDS patients treated with HFNO is limited. The only previously published study included 20 patients of which 9 patients (45%) required intubation; for the 11 non-intubated patients, 8 received HFNO + awake PP, and six of them needed escalation to NIV [24].

Data on the use of awake-PP in COVID-19 patients is limited to small, single-center studies or case series with contradictory results. Elharrar et al. [11] examined the effects of awake-PP in 24 patients receiving oxygen therapy. Oxygenation improved in about one fourth of patients and deteriorated again after turning the patient to supine. No information regarding the need for intubation was provided [11]. Thompson et al. [12] in a similar population of 25 patients managed with conventional oxygen therapy found a heterogeneous response to awake-PP with improvements in SpO_2_ ranging from 1 to 37%, but 12 patients (48%) patients required intubation. Better results were found by Ng et al. [13] who applied daily awake-PP sessions of 5 h in 10 non-ICU patients with only one needing intubation. Similar results were reported by Sartini et al. [14] in 15 non-ICU patients supported with NIV in whom awake-PP was used as a rescue therapy, resulting in an improvement of oxygenation and respiratory rate, and only one patient required intubation. In the study by Xu et al. [15], intubation was needed in 5 (50%) out of 10 patients managed with HFNO plus early awake-PP 16 h/day during three consecutive days. Finally, Coppo et al. [16] performed a feasibility and physiological study including 56 patients in which awake prone lasting > 3 h improved oxygenation but not dyspnea and respiratory rate. Similar to previous studies, this improvement in oxygenation was maintained only in half of their patients after returning to the supine position. Of note, awake-PP was applied earlier (median of 1.9 days) in responders. However, no differences in the need for intubation were found between responders and non-responders (26% vs. 30%) [16]. Those previous reports together with our current study do not support the use of awake-PP as an effective adjunctive strategy for preventing intubation.

As oxygenation is generally improved on awake-PP, one potential risk would be an undue delay in intubation which could potentially worsen prognosis, as demonstrated in previous studies in non-COVID-19 patients [8]. Coppo et al. [16] did not find any differences in time to intubation between responders and non-responders to awake-PP in their cohort of COVID-19 patients. Our original and adjusted data show that patients in the HFNO + awake-PP group had a strong trend toward a delay in intubation of 2 days; however, 28-day mortality was similar in both treatment groups.

This study has several strengths. First, to date, it is the largest study including 199 patients from 36 intensive care units. Second, this multicenter nationwide prospective daily data collection protocol provided a very detailed description of the patient course during the study period. Third, to the best of our knowledge, this is the first study that prospectively explored the association between awake-PP and the risk for intubation in original and adjusted COVID-19 samples with severe hypoxemic ARF. However, we acknowledge some limitations. First, we were unable to determine whether clinicians used awake-PP as usual practice for COVID-19 patients or as a rescue strategy. Second, as in our case report form, prone was only considered when it was applied for > 16 h/day, we cannot extend our results to patients pronated for shorter periods of time. Whether awake prone position for less than 16 h/day could have reduced the risk of intubation is not available from our data. The patients in this group may have acted as an uncontrolled confounder minimizing the differences between groups. This should be further investigated in a randomized controlled trial. Third, intubation criteria were not uniformly defined and protocolized, which may limit the generalizability of our results. Fourth, although we controlled for variables describing patient’s severity, we acknowledge that despite our efforts to control for this possible source of bias, there is a risk of residual confounding or unrecognized biases. Fifth, due to the nature of the database, the sample size was not calculated and therefore the number of patients included in this analysis could be less than necessary to have adequate power for the primary endpoint. However, an ongoing RCT (NCT04347941) includes a total of 200 patients, which is very similar to our 199 patients, to demonstrate the effects of awake prone position on intubation in COVID-19 patients with ARF. Finally, due to the pragmatic nature of our data collection, variables such as SpO_2_, PaO_2_/FiO_2_, RR, or ROX index were not collected before and after awake-PP sessions. Therefore, individual responses could not be determined, limiting the possibility of analyzing the effects of prone on intubation in specific subpopulations of patients. Nevertheless, current data showed that responders, defined as those patients that improved oxygenation when managed with HFNO and awake-PP, did not decrease their risk for intubation.

## Conclusions

To the best of our knowledge, this is the first multicenter study that prospectively evaluated the benefits and the role of HFNO combined with awake prone positioning in the prevention of intubation in an adjusted large cohort of COVID-19 patients. We found that this combined approach did not reduce the risk of intubation, but could increase the risk of delaying intubation. In the current study, awake-PP did not affect 28-day mortality. The interpretation of these results may be limited by the observational design, and therefore future studies are needed to identify potential subpopulations that may benefit from awake prone positioning in COVID-19 patients with acute hypoxemic respiratory failure.

## Supplementary information

**Additional file 1: Table 1.** Baseline characteristics of patients with HFNO before and after adjustment. **Table 2.** Clinical evolution of patients with HFNO (maximum or minimum values) before and after adjustment. **Table 3.** Baseline characteristics of patients with HFNO plus awake prone positioning before and after adjustment. **Table 4.** Clinical evolution (maximum or minimum values) of patients with HFNO plus awake prone position before and after adjustment. **Table 5.** Outcomes of the original-eligible population and weighted population. **Figure 1.** Peripheral oxyhemoglobin saturation (%) over time in the adjusted population. **Figure 2.** Respiratory rate (breath per minute) over time in the adjusted population. **Figure 3.** ROX Index [(SpO_2_/FiO_2_) / Respiratory rate] over time in the adjusted population. **Figure 4.** Probability of being intubated in patients stratified by PaO_2_/FiO_2_. **Figure 5.** Probability of 28-day mortality in patients stratified by PaO_2_/FiO_2_.

## Data Availability

The datasets used and/or analyzed during the current study are available from the corresponding author on reasonable request.

## Abbreviations

APACHE: Acute physiological and Chronic Health disease Classification System
ARDS: Acute respiratory distress syndrome
ARF: Acute respiratory failure
Awake-PP: Awake prone positioning
BMI: Body mass index
COVID-19: Coronavirus disease 19
CRP: C-reactive protein
HFNO: High-flow nasal oxygen therapy
ICU: Intensive care unit
IQR: Interquartile range
LOS: Length of stay
MV: Mechanical ventilation
NIV: Non-invasive ventilation
PaO2/FiO2: Partial pressure of arterial oxygen to inspiratory oxygen fraction ratio
P-SILI: Patient self-inflicted lung injury
RR: Respiratory rate
SOFA: Sequential Organ Failure Assessment
SpO2: Peripheral oxyhemoglobin saturation
STROBE: Strengthening the Reporting of Observational Studies in Epidemiology
V/Q: Ventilation-perfusion
WHO: World Health Organization
