# Supplementary material for: Awake prone positioning does not reduce the risk of intubation in COVID-19 treated with high-flow nasal oxygen therapy: a multicenter, adjusted cohort study
Source: Crit Care. 2020 Oct 6;24:597. doi: 10.1186/s13054-020-03314-6 (PMC7537953; doi:10.1186/s13054-020-03314-6)
Supplement: Supplementary file 1 — Additional file 1: Table 1. Baseline characteristics of patients with HFNO before and after adjustment. Table 2. Clinical evolution of patients with HFNO (maximum or minimum values) before and after adjustment. Table 3. Baseline characteristics of patients with HFNO plus awake prone positioning before and after adjustment. Table 4. Clinical evolution (maximum or minimum values) of patients with HFNO plus awake prone position before and after adjustment. Table 5. Outcomes of the original-eligible population and weighted population. Figure 1. Peripheral oxyhemoglobin saturation (%) over time in the adjusted population. Figure 2. Respiratory rate (breath per minute) over time in the adjusted population. Figure 3. ROX Index [(SpO2/FiO2) / Respiratory rate] over time in the adjusted population. Figure 4. Probability of being intubated in patients stratified by PaO2/FiO2. Figure 5. Probability of 28-day mortality in patients stratified by PaO2/FiO2. [file 13054_2020_3314_MOESM1_ESM.docx]

**Electronic Supplement**

**Awake prone positioning does not reduce the risk of intubation in COVID-19 treated with High-Flow Nasal Oxygen therapy.**

**A multicenter, adjusted cohort study**

**Authors:** Ferrando C, Mellado-Artigas R, Gea A, et al.

**Table 1. Baseline characteristics of patients with HFNO before and after adjustment.**

|  | **Original sample** | | **Weighted sample** | |
| --- | --- | --- | --- | --- |
|  | **Non-intubated**  **(n=117)** | **Intubated**  **(n=82)** | **Non-intubated**  **64.27%** | **Intubated**  **35.73%** |
| **Patients demographics and comorbidities** | | | | |
| Age | 62.0 [55.0-68.0] /117 | 63.0 [55.0-71.0] /82 | 59.4 | 62.5 |
| Gender, female | 29/117 (24.8%) | 23/80 (28.7%) | 31.7% | 28.0.% |
| Body mass index, kg/m^2^ | 26.7 [24.9-30.5] /98 | 27.8 [25.0-29.4] /71 | 28.5 | 28.5 |
| Arterial Hypertension | 41/117 (35.0%) | 39/82 (47.6%) | 34.8% | 49.6% |
| Diabetes Mellitus | 17/117 (14.5%) | 15/82 (18.3%) | 11.9% | 22.7% |
| Chronic cardiac failure | 4/117 (3.4%) | 0/82 (0.0%) | 4.0% | 0.0% |
| Chronic renal failure | 10/117 (8.5%) | 8/82 (9.8%) | 7.6% | 3.99% |
| Asthma | 5/117 (4.3%) | 1/82 (1.2%) | 10.0% | 1.91% |
| COPD | 5/117 (4.3%) | 5/82 (6.1%) | 4.5% | 7.09% |
| Obesity | 26/98 (26.5%) | 16/71 (22.5%) | 34.0% | 25.4% |
| Dyslipidemia | 7/117 (5.9%) | 12/82 (14.6%) | 4.8% | 10.8% |
| Malignancy | 10/117 (8.5%) | 2/82 (2.4%) | 5.8% | 1.8% |
| **Medical treatment** | | | | |
| Antihypertensives | 44/117 (37.6%) | 37/82 (45.1%) | 39.7% | 44.4% |
| Hypoglycemic agents | 12/117 (10.3%) | 13/82 (15.8%) | 10.5% | 30.3% |
| Antiplatelet agents | 15/117 (12.8%) | 7/82 (8.5%) | 11.3% | 7.7% |
| Anticoagulants | 8/117 (6.8%) | 3/82 (3.7%) | 8.6% | 6.0% |
| Bronchodilators | 25/117 (21.4%) | 20/82 (24.4%) | 22.3% | 23.3% |
| Lipid lowering agents | 5/117 (4.3%) | 6/82 (7.3%) | 6.7% | 5.7% |
| Thyroid hormone replacement | 11/117 (9.4%) | 8/82 (9.8%) | 16.4% | 16.7% |
| Immunossupressors | 5/117 (4.3%) | 5/82 (6.1%) | 3.4% | 1.8% |
| Corticosteroids | 5/117 (4.3%) | 6/82 (7.3%) | 3.4% | 1.8% |
| **Chronology** | | | | |
| Time from symptoms onset to hospital admission | 7.0 [5.0-10.0] /115 | 7.0 [3.0-9.0] /81 | 7.7 | 7.1 |
| Time from symptoms onset to HFNO | 11.0 [9.0-13.5] /116 | 10.0 [6.0-12.0] /81 | 10.4 | 9.5 |
| **Symptoms at ICU admission** | | | | |
| Fever | 105/117 (89.7%) | 67/82 (81.7%) | 90.2% | 83.9% |
| Cough | 83/117 (70.9%) | 47/82 (57.3%) | 73.3% | 55.7% |
| Dyspnoea | 71/117 (60.7%) | 60/82 (73.2%) | 60.6% | 75.7% |
| Malaise | 50/117 (42.7%) | 34/82 (41.5%) | 42.9% | 53.2% |
| Myalgia | 20/117 (17.1%) | 12/82 (14.6%) | 17.7% | 19.1% |
| Headache | 11/117 (9.4%) | 7/82 (8.5%) | 8.0% | 5.5% |
| Rhinorrhea | 1/117 (0.8%) | 1/82 (1.2%) | 1.2% | 2.9% |
| Vomiting | 7/117 (5.9%) | 7/82 (8.5%) | 6.9% | 3.3% |
| Arthralgia | 7/117 (5.9%) | 3/82 (3.7%) | 5.2% | 1.9% |
| Chest pain | 10/117 (8.5%) | 3/82 (3.7%) | 7.4% | 4.1% |
| Increased sputum | 12/117 (10.3%) | 8/82 (9.8%) | 10.8% | 4.8% |
| Anosmia | 10/117 (8.5%) | 0/82 (0.0%) | 10.0% | 0.0% |
| Pharyngodynia | 5/117 (4.3%) | 1/82 (1.2%) | 3.7% | 1.0% |
| Diarrhea | 16/117 (13.7%) | 13/82 (15.8%) | 15.2% | 16.2% |
| Fatigue | 2/117 (1.7%) | 3/82 (3.7%) | 0.8% | 4.3% |
| **Scores** | | | | |
| APACHE II | 10.0 [6.0-13.0] /94 | 12.0 [9.0-15.0] /59 | 10 | 12 |
| Non-respiratory SOFA | 4.0 [4.0-4.0] /99 | 4.0 [4.0-6.0] /63 | 4.4 | 5.1 |
| **Vital Signs** | | | | |
| Temperature, ºC | 36.8 [36.1-37.4] /113 | 37.0 [36.1-37.8] /82 | 36.8 | 36.9 |
| Mean arterial pressure, mmHg | 86.7 [80.0-93.3] /114 | 86.7 [78.7-96.7] /82 | 87.5 | 86.4 |
| Heart rate, bpm | 76.5 [70.0-90.0] /114 | 85.0 [78.0-93.0] /81 | 79.8 | 84.1 |
| SpO_2_, % | 90.5 [89.0-94.0] /114 | 89.0 [86.0-92.0] /81 | 90.7 | 89.7 |
| Respiratory rate, bpm | 24.0 [20.0-28.0] /113 | 26.0 [23.0-32.0] /77 | 24.5 | 27.7 |
| **Arterial blood gas** | | | | |
| PaO_2_/FiO_2_ | 125.0 [93.0-164.0] /101 | 99.5 [82.0-144.0] /74 | 134.0 | 127.5 |
| PaCO_2_, mmHg | 34.2 [31.0-37.3] /105 | 34.0 [29.0-38.0] /75 | 34.8 | 33.8 |
| **Laboratory findings** | | | | |
| Ferritin, ng/mL | 990 [573-1857] /79 | 1394 [798-2302] /49 | 1478 | 2074 |
| D-Dimer, ng/mL | 900 [565-1672] /99 | 1070 [572-2139] /63 | 1458 | 1916 |
| CRP, mg/dL | 15.9 [8.32-40.53] /108 | 19.3 [9.12-53.48] /76 | 57.6 | 55.4 |
| Lymphocytes, 10^3^/μL | 0.62 [0.40-0.90] /109 | 0.60 [0.40-0.82] /76 | 0.83 | 0.69 |
| IL-6, pg/mL | 132.0 [47.0-202.0] /15 | 93.0 [42.0-334.0] /13 | 186.3 | 123.4 |
| LDH, U/L | 372 [311-450] /107 | 450.0 [352.0-597.0] /69 | 366.8 | 530.4 |
| Leukocytes, 10^3^/μL | 7.1 [5.2-10.9] /108 | 6.7 [4.3-9.2] /75 | 8.1 | 6.7 |
| Procalcitonin, ng/mL | 0.17 [0.1-0.3] /80 | 0.23 [0.1-0.6] /58 | 0.49 | 0.67 |
| Platelets, 1000/mm^3^ | 255.0 [181.0-359.0] /109 | 190.0 [135.0-264.0] /77 | 272.7 | 206.8 |
| Bilirrubin, mg/dL | 0.70 [0.5-1.0] /102 | 0.59 [0.4-0.8] /70 | 0.79 | 0.86 |
| GPT, U/L | 42.0 [24.0-86.0] /106 | 37.9 [22.9-69.9] /76 | 60.2 | 72.4 |
| Creatinine, mg/dL | 0.81 [0.6-0.9] /108 | 0.86 [0.7-1.1] /76 | 0.93 | 1.11 |
| Urea, mg/dL | 34.4 [25.0-54.0] /64 | 36.0 [25.0-48.0] /54 | 44.6 | 36.3 |
| Troponin, ng/mL | 9.5 [2.8-20.1] /60 | 13.5 [4.9-23.8] /42 | 11.0 | 22.6 |
| NTproBNP, pg/mL | 464.0 [91.0-1451.0] /17 | 261.0 [152.5-372.0] /5 | 777.9 | 686.7 |
| Hematocrite, % | 39.0 [35.1-42.9] /108 | 38.2 [35.0-42.5] /68 | 39.5 | 37.9 |
| Lactate, mmol/L | 1.6 [1.2-2.0] /69 | 1.3 [1.1-1.9] /46 | 1.8 | 1.8 |

Values were obtained from each patient on day 1 of HFNO. Categorical variables are expressed as proportion, and continuous variables as median (IQR) for original-eligible population and percentage and mean for weighted population. Abbreviations. HFNO: high flow nasal oxygen therapy; COPD: chronic obstructive pulmonary disease; SOFA: sequential organ failure assessment; RCP: C-reactive protein; IL: interleukin; LDH: lactate dehydrogenase; GPT: Glutamate pyruvate transaminase.

**Table 2. Clinical evolution of patients with HFNO (maximum or minimum values) before and after adjustment.**

|  | **Original sample** | | **Weighted sample** | |
| --- | --- | --- | --- | --- |
|  | **Non-intubated**  **(n=117)** | **Intubated**  **(n=82)** | **Non-intubated**  **64.27%** | **Intubated**  **35.73%** |
| **Scores** | | | | |
| Non-respiratory SOFA | 4.0 [4.0-4.5] /104 | 5.0 [4.0-7.0] /67 | 4.5 | 5.6 |
| **Vital Signs** | | | | |
| Temperature, ºC | 37.0 [36.5-37.7] /113 | 37.4 [36.5-38.0] /82 | 37.1 | 37.3 |
| Mean arterial pressure, mmHg | 78.7 [73.3-84.7] /113 | 74.3 [66.7-82.0] /81 | 78.3 | 73.1 |
| Heart rate, bpm | 84.0 [75.0-95.0] /113 | 89.5 [80.0-99.0] /82 | 86.3 | 92.7 |
| SpO_2_, % | 90.0 [87.0-92.0] /114 | 87.0 [83.0-90.0] /81 | 89.1 | 87.2 |
| Respiratory rate minimum, bpm | 20.0 [17.0-23.0] /113 | 22.0 [19.0-25.0] /82 | 19.9 | 21.5 |
| Respiratory rate, maximum bpm | 25.0 [23.0-30.0] /113 | 28.5 [25.0-35.0] /82 | 26.2 | 29.8 |
| **Arterial blood gas** | | | | |
| PaO_2_/FiO_2_ | 104.0 [81.0-133.0] /102 | 88.0 [76.0-110.0] /79 | 117.9 | 98.9 |
| PaCO_2_, mmHg | 39.0 [34.1-42.0] /105 | 46.0 [37.0-55.0] /79 | 40.3 | 50.7 |
| **Laboratory findings** | | | | |
| Ferritin, ng/mL | 1222 [731-1979] /95 | 1650 [766-2955] /57 | 1722 | 2131 |
| D- Dimer, ng/mL | 1768 [1050-4135] /104 | 1112 [700-3835] /68 | 2926 | 2375 |
| CRP, mg/dL | 20.4 [8.5-42.0] /108 | 24.1 [12.1-74.0] /77 | 60.9 | 65.2 |
| Lymphocytes, 10^3^/μL | 0.46 [0.3-0.7] /111 | 0.45 [0.3-0.7] /77 | 0.5 | 0.5 |
| IL-6, pg/mL | 166.5 [42.8-358.5] /16 | 61.8 [35.6-578.0] /15 | 711.2 | 252.9 |
| LDH, U/L | 394.7 [329.0-482.0] /106 | 506.0 [383.0-664.0] /70 | 407.7 | 570.8 |
| Leukocytes, 10^3^/μL | 8.8 [5.9-12.6] /103 | 7.6 [5.3-11.3] /70 | 9.8 | 8.9 |
| Procalcitonin, ng/mL | 0.18 [0.1-0.4] /96 | 0.25 [0.1-0.6] /63 | 0.5 | 1.7 |
| Platelets, 1000/mm^3^ | 351.0 [260.0-436.0] /111 | 241.5 [171.0-353.0] /78 | 368.4 | 261.8 |
| Bilirrubin, mg/dL | 0.8 [0.6-1.3] /107 | 0.7 [0.5-1.0] /73 | 1.1 | 1.1 |
| GPT, U/L | 70.5 [32.0-121.0] /110 | 51.0 [26.0-86.0] /78 | 96.6 | 83.0 |
| Creatinine, mg/dL | 0.85 [0.68-1.04] /111 | 0.97 [0.74-1.26] /77 | 1.00 | 1.22 |
| Urea, mg/dL | 40.0 [28.0-61.9] /81 | 43.2 [30.5-63.0] /60 | 50.1 | 45.6 |
| Troponin, ng/mL | 9.4 [3.8-19.9] /81 | 15.8 [8.0-40.3] /47 | 30.1 | 26.8 |
| NTproBNP, pg/mL | 258.1 [91.0-1080.0] /22 | 343.5 [205.5-892.8] /12 | 680.6 | 733.7 |
| Hematocrite, % | 39.0 [35.3-42.0] /93 | 38.0 [34.0-41.0] /63 | 39.0 | 37.7 |
| Lactate, mmol/L | 1.6 [1.2-2.1] /60 | 1.3 [1.1-2.0] /48 | 1.8 | 1.9 |

Maximum or minimum values during the period of HFNO. Categorical variables are expressed as proportion, and continuous variables as median (IQR) for original-eligible population and percentage and mean for weighted population. Abbreviations. HFNO: high flow nasal oxygen therapy; SpO_2_: peripheral oxyhemoglobin saturation; SOFA: sequential organ failure assessment; RCP: C-reactive protein; IL: interleukin; LDH: lactate dehydrogenase; GPT: Glutamate pyruvate transaminase.

**Table 3. Baseline characteristics of patients with HFNO plus awake prone positioning before and after adjustment.**

|  | **Original sample** | | **Weighted sample** | |
| --- | --- | --- | --- | --- |
|  | **Non-intubated**  **(n=33)** | **Intubated**  **(n=22)** | **Non-intubated**  **62.66%** | **Intubated**  **37.34%** |
| **Patients demographics and comorbidities** | | | | |
| Age | 60.0 [55.0-67.0] /33 | 63.5 [53.0-71.0] /22 | 60.2 | 62.0 |
| Gender, female | 8/33 (24.2%) | 5/21 (23.8%) | 37.9% | 27.3% |
| Body mass index, kg/m^2^ | 26.3 [24.9-32.5] /28 | 27.3 [24.5-31.1] /21 | 28.6 | 27.6 |
| Arterial Hypertension | 10/33 (30.3%) | 10/22 (45.4%) | 27.5% | 45.5% |
| Diabetes Mellitus | 6/33 (18.2%) | 3/22 (13.6%) | 14.7% | 3.9% |
| Chronic cardiac failure | 2/33 (6.1%) | 0/22 (0.0%) | 8.3% | 0% |
| Chronic renal failure | 3/33 (9.1%) | 1/22 (4.5%) | 9.9% | 0% |
| Asthma | 1/33 (3.0%) | 0/22 (0.0%) | 9.9% | 0% |
| COPD | 2/33 (6.1%) | 2/22 (9.1%) | 7.7% | 8.8% |
| Obesity | 11/28 (39.3%) | 6/21 (28.6%) | 38.5% | 22.3% |
| Dyslipidemia | 1/33 (3.0%) | 3/22 (13.6%) | 2.8% | 7.5% |
| Malignancy | 2/33 (6.1%) | 1/22 (4.5%) | 5.2% | 0% |
| **Medical treatment** | | | | |
| Antihypertensives | 8/33 (24.2%) | 11/22 (50.0%) | 26.4% | 51.8% |
| Hypoglycemic agents | 4/33 (12.1%) | 3/22 (13.6%) | 10.4% | 28.2% |
| Antiplatelet agents | 3/33 (9.1%) | 2/22 (9.1%) | 10.4% | 16.8% |
| Anticoagulants | 1/33 (3.0%) | 0/22 (0.0%) | 1.9% | 0% |
| Bronchodilators | 5/33 (15.1%) | 5/22 (22.7%) | 21.4% | 26.4% |
| Lipid lowering agents | 1/33 (3.0%) | 2/22 (9.1%) | 5.1% | 0% |
| Thyroid hormone replacement | 4/33 (12.1%) | 5/22 (22.7%) | 20.1% | 33.1% |
| Immunossupressors | 0/33 (0.0%) | 1/22 (4.5%) | 0% | 0% |
| Corticosteroids | 0/33 (0.0%) | 2/22 (9.1%) | 0% | 0% |
| **Chronology** | | | | |
| Time from symptoms onset to hospital admission | 7.0 [5.0-10.0] /33 | 7.5 [3.0-10.0] /22 | 7.7 | 7.6 |
| Time from symptoms onset to HFNO | 11.0 [9.0-12.0] /33 | 10.5 [8.0-13.0] /22 | 9.9 | 10.6 |
| **Symptoms at ICU admission** | | | | |
| Fever | 32/33 (96.9%) | 19/22 (86.4%) | 97.3% | 77.6% |
| Cough | 22/33 (66.7%) | 14/22 (63.6%) | 69.9% | 49.2% |
| Dyspnoea | 21/33 (63.6%) | 18/22 (81.8%) | 67.6% | 84.3% |
| Malaise | 18/33 (54.5%) | 9/22 (40.9%) | 56.1% | 56.8% |
| Myalgia | 6/33 (18.2%) | 4/22 (18.2%) | 15.8% | 23.6% |
| Headache | 5/33 (15.1%) | 1/22 (4.5%) | 7.3% | 3.2% |
| Rhinorrhea | 0/33 (0.0%) | 1/22 (4.5%) | 0% | 8.8% |
| Vomiting | 2/33 (6.1%) | 2/22 (9.2%) | 10.2% | 3.9% |
| Arthralgia | 3/33 (9.1%) | 1/22 (4.5%) | 8.7% | 0% |
| Chest pain | 0/33 (0.0%) | 1/22 (4.5%) | 0% | 0% |
| Increased sputum | 3/33 (9.1%) | 3/22 (13.6%) | 8.7% | 14.6% |
| Anosmia | 4/33 (12.1%) | 0/22 (0.0%) | 10.3% | 0% |
| Pharyngodynia | 0/33 (0.0%) | 1/22 (4.5%) | 0% | 3.2% |
| Diarrhea | 6/33 (18.2%) | 3/22 (13.6%) | 18.2% | 9.5% |
| Fatigue | 1/33 (3.0%) | 3/22 (13.6%) | 2.6% | 13.1% |
| **Scores** | | | | |
| APACHE II | 8.0 [5.0-11.0] /30 | 13.0 [8.0-18.7] /16 | 8.9 | 14.4 |
| Non-respiratory SOFA | 4.0 [4.0-4.0] /30 | 4.0 [4.0-4.0] /16 | 4.7 | 4.6 |
| **Vital Signs** | | | | |
| Temperature, ºC | 36.8 [36.3-37.4] /32 | 36.6 [36.0-37.3] /22 | 37.0 | 36.6 |
| Mean arterial pressure, mmHg | 86.8 [79.0-91.7] /32 | 83.0 [70.7-93.3] /22 | 84.1 | 80.9 |
| Heart rate, bpm | 71.0 [65.0-82.0] /32 | 80.5 [71.0-96.0] /22 | 75.9 | 83.9 |
| SpO_2_, % | 90.0 [89.0-92.5] /32 | 89.0 [86.0-92.0] /22 | 90.1 | 90.8 |
| Respiratory rate, bpm | 22.0 [20.0-27.0] /32 | 27.0 [20.0-32.0] /22 | 24.5 | 27.3 |
| **Arterial blood gas** | | | | |
| PaO_2_/FiO_2_ | 125.0 [104.5-178.5] /32 | 120.0 [84.0-219.0] /19 | 139.5 | 162.9 |
| PaCO_2_, mmHg | 35.3 [30.8-38.0] /32 | 34.5 [30.0-39.7] /19 | 33.9 | 33.9 |
| **Laboratory findings** | | | | |
| Ferritin, ng/mL | 787 [573-985] /24 | 1832 [987-3422] /17 | 1099 | 2743 |
| D- Dimer, ng/mL | 895 [557-1700] /30 | 1090 [540-2250]/18 | 1629 | 1574 |
| CRP, mg/dL | 14.6 [8.4-124.1] /32 | 36.0 [9.5-167.0] /21 | 48.2 | 73.7 |
| Lymphocytes, 10^3^/μL | 0.7 [0.4-1.0] /32 | 0.58 [0.4-0.7] /21 | 0.8 | 0.56 |
| IL-6, pg/mL | 119.0 [43.0-132.0] /5 | 67.5 [35.5-578.0] /6 | 131.0 | 137.6 |
| LDH, U/L | 361.0 [309.0-412.0] /31 | 511.0 [348.0-617.5] /20 | 382.2 | 518.9 |
| Leukocytes, 10^3^/μL | 6.5 [4.3-8.6] /32 | 6.6 [4.5-9.7] /20 | 7.0 | 6.1 |
| Procalcitonin, ng/mL | 0.12 [0.1-0.3] /24 | 0.18 [0.1-1.7] /15 | 0.26 | 0.36 |
| Platelets, 1000/mm^3^ | 239.5 [168.5-300.5] /32 | 231.0 [131.0-264.0] /21 | 234.8 | 198.7 |
| Bilirrubin, mg/dL | 0.7 [0.5-1.1] /30 | 0.6 [0.4-0.8] /18 | 0.72 | 0.69 |
| GPT, U/L | 38.0 [26.0-94.0] /31 | 35.0 [22.0-62.0] /21 | 64.4 | 59.6 |
| Creatinine, mg/dL | 0.8 [0.6-0.9] /32 | 0.8 [0.7-1.0] /20 | 1.0 | 1.1 |
| Urea, mg/dL | 30.0 [19.5-45.0] /24 | 43.5 [21.0-62.0] /18 | 35.0 | 32.2 |
| Troponin, ng/mL | 7.04 [2.8-13.0] /21 | 11.0 [2.7-34.7] /12 | 7.9 | 19.9 |
| NTproBNP, pg/mL | 91.0 [50.0-360.0] /5 | 1263.0 [1263.0-1263.0]/1 | 320.5 | 1263.0 |
| Hematocrite, % | 42.0 [35.8-44.0] /32 | 39.5 [38.0-42.0] /18 | 39.9 | 38.4 |
| Lactate, mmol/L | 1.7 [1.3-2.0] /21 | 1.4 [1.2-1.8] /12 | 1.9 | 1.7 |

Values were obtained from each patient on day 1 of HFNO+awake-PP. Categorical variables are expressed as proportion, and continuous variables as median (IQR) for original-eligible population and percentage and mean for weighted population. Abbreviations. HFNO: high flow nasal oxygen therapy; COPD: chronic obstructive pulmonary disease; SOFA: sequential organ failure assessment; RCP: C-reactive protein; IL: interleukin; LDH: lactate dehydrogenase; GPT: Glutamate pyruvate transaminase.

**Table 4. Clinical evolution (maximum or minimum values) of patients with HFNO plus awake prone position before and after adjustment.**

|  | **Original sample** | | **Weighted sample** | |
| --- | --- | --- | --- | --- |
|  | **Non-intubated**  **(n=33)** | **Intubated**  **(n=22)** | **Non-intubated**  **62.66%** | **Intubated**  **37.34%** |
| **Scores** | | | | |
| Non-respiratory SOFA | 4.0 [4.0-4.0] /30 | 4.0 [4.0-7.0] /16 | 4.7 | 5.5 |
| **Vital Signs** | | | | |
| Temperature, ºC | 36.9 [36.7-38.1] /32 | 37.2 [36.5-37.8] /22 | 37.4 | 37.0 |
| Mean arterial pressure, mmHg | 76.7 [72.3-82.0] /32 | 73.5 [66.7-86.7] /22 | 75.2 | 70.3 |
| Heart rate, bpm | 83.0 [73.5-99.0] /32 | 86.5 [80.0-103.0] /22 | 90.0 | 93.8 |
| SpO_2_, % | 89.0 [86.5-91.5] /32 | 86.5 [82.0-88.0] /22 | 87.6 | 87.6 |
| Respiratory rate minimum, bpm | 18.0 [15.5-21.0] /32 | 20.0 [17.0-24.0] /22 | 19.2 | 20.5 |
| Respiratory rate, maximum bpm | 25.0 [22.5-28.5] /32 | 29.0 [26.0-33.0] /22 | 25.7 | 29.5 |
| **Arterial blood gas** | | | | |
| PaO_2_/FiO_2_ | 109.5 [87.0-130.5] /32 | 86.0 [68.0-111.0] /21 | 123.8 | 97.2 |
| PaCO_2_, mmHg | 40.3 [35.2-42.3] /32 | 47.0 [38.0-53.6] /21 | 39.0 | 48.2 |
| **Laboratory findings** | | | | |
| Ferritin, ng/mL | 1027 [751-2125] /27 | 1962 [987-3422] /18 | 1477 | 2704 |
| D- Dimer, ng/mL | 1880 [1200-4000]/31 | 1080 [800-2400] /19 | 3209 | 1457 |
| CRP, mg/dL | 20.6 [8.5-124.1] /32 | 36.0 [14.7-178.0] /21 | 52.2 | 80.0 |
| Lymphocytes, 10e^3^/μL | 0.51 [0.3-0.7] /32 | 0.40 [0.2-0.5] /21 | 0.48 | 0.31 |
| IL-6, pg/mL | 43.0 [9.5-156.0] /7 | 142.9 [24.0-932.0] /7 | 96.6 | 402.2 |
| LDH, U/L | 413.0 [352.0-493.0] /31 | 560.5 [369.0-722.5] /20 | 455.7 | 547.6 |
| Leukocytes, 10^3^/μL | 7.8 [5.6-12.5] /32 | 7.7 [4.2-11.3] /19 | 9.7 | 7.8 |
| Procalcitonin, ng/mL | 0.17 [0.0-0.3] /29 | 0.29 [0.1-1.1] /16 | 0.24 | 0.51 |
| Platelets, 1000/mm^3^ | 335.0 [292.0-401.0] /32 | 255.0 [184.0-311.0] /21 | 369.9 | 262.3 |
| Bilirrubin, mg/dL | 0.9 [0.6-1.3] /31 | 0.8 [0.6-1.0] /19 | 0.8 | 0.9 |
| GPT, U/L | 79.0 [32.0-159.5] /32 | 40.0 [26.0-62.0] /21 | 127.1 | 69.6 |
| Creatinine, mg/dL | 0.83 [0.7-0.9] /32 | 0.97 [0.8-1.2] /20 | 1.07 | 1.14 |
| Urea, mg/dL | 32.0 [26.0-56.0] /31 | 47.5 [33.8-67.0] /19 | 40.7 | 45.9 |
| Troponin, ng/mL | 9.0 [5.3-18.1] /26 | 10.0 [4.6-40.3] /13 | 62.9 | 25.2 |
| NTproBNP, pg/mL | 158.0 [33.0-685.6] /8 | 761.0 [315.0-1019.0] /6 | 380.5 | 864.4 |
| Hematocrite, % | 41.4 [35.8-43.5] /28 | 38.5 [38.0-39.9] /17 | 39.8 | 38.7 |
| Lactate, mmol/L | 1.5 [1.2-2.1] /18 | 1.4 [1.3-1.9] /13 | 1.9 | 1.8 |

Maximum or minimum values during the period of HFNO. Categorical variables are expressed as proportion, and continuous variables as median (IQR) for original-eligible population and percentage and mean for weighted population. Abbreviations. HFNO: high flow nasal oxygen therapy; SpO_2_: peripheral oxyhemoglobin saturation; SOFA: sequential organ failure assessment; RCP: C-reactive protein; IL: interleukin; LDH: lactate dehydrogenase; GPT: Glutamate pyruvate transaminase.

**Table 5. Outcomes of the original-eligible population and weighted population.**

| Outcome | **Original sample** | | | **Weighted sample** | | |
| --- | --- | --- | --- | --- | --- | --- |
|  | HFNO  (n=144) | HFNO+awake-PP  (n=55) | p-value | HFNO  67.4% | HFNO+awake-PP  32.5% | p-value |
| Intubated | 60 (41.7%) | 22 (40.0%) | 0.481 | 35.0% | 37.3% | 0.824 |
| Days from HFNO start to intubation* | 1 [1-2.5] | 2 [1-3] | 0.055 | 2.05 | 4.18 | 0.054 |
| ICU length of stay** | 7.5 [4-14] | 8 [5-14] | 0.276 | 11.6 | 11.4 | 0.950 |
| ICU length of stay of discharge patients** | 7 [4-12] | 8 [5-13] | 0.417 | 10.5 | 9.3 | 0.472 |
| Discharge from ICU** | 105/122 (86.1%) | 41/49 (83.7%) | 0.427 | 90.8% | 80.0% | 0.18 |
| ICU mortality** | 17/122 (13.9%) | 8/49 (16.3% |  | 9.2% | 20.0% |  |

Categorical variables are expressed as proportion, and continuous variables as median (IQR) for original-eligible population and percentage and mean for weighted population. P-value cut-off at 0.05. * Intubated patients, **Excluding patients who are in ICU. Abbreviations. HFNO: high flow nasal oxygen therapy; ICU: intensive care unit.

**Figure 1. Peripheral oxyhemoglobin saturation (%) over time in the adjusted population.**


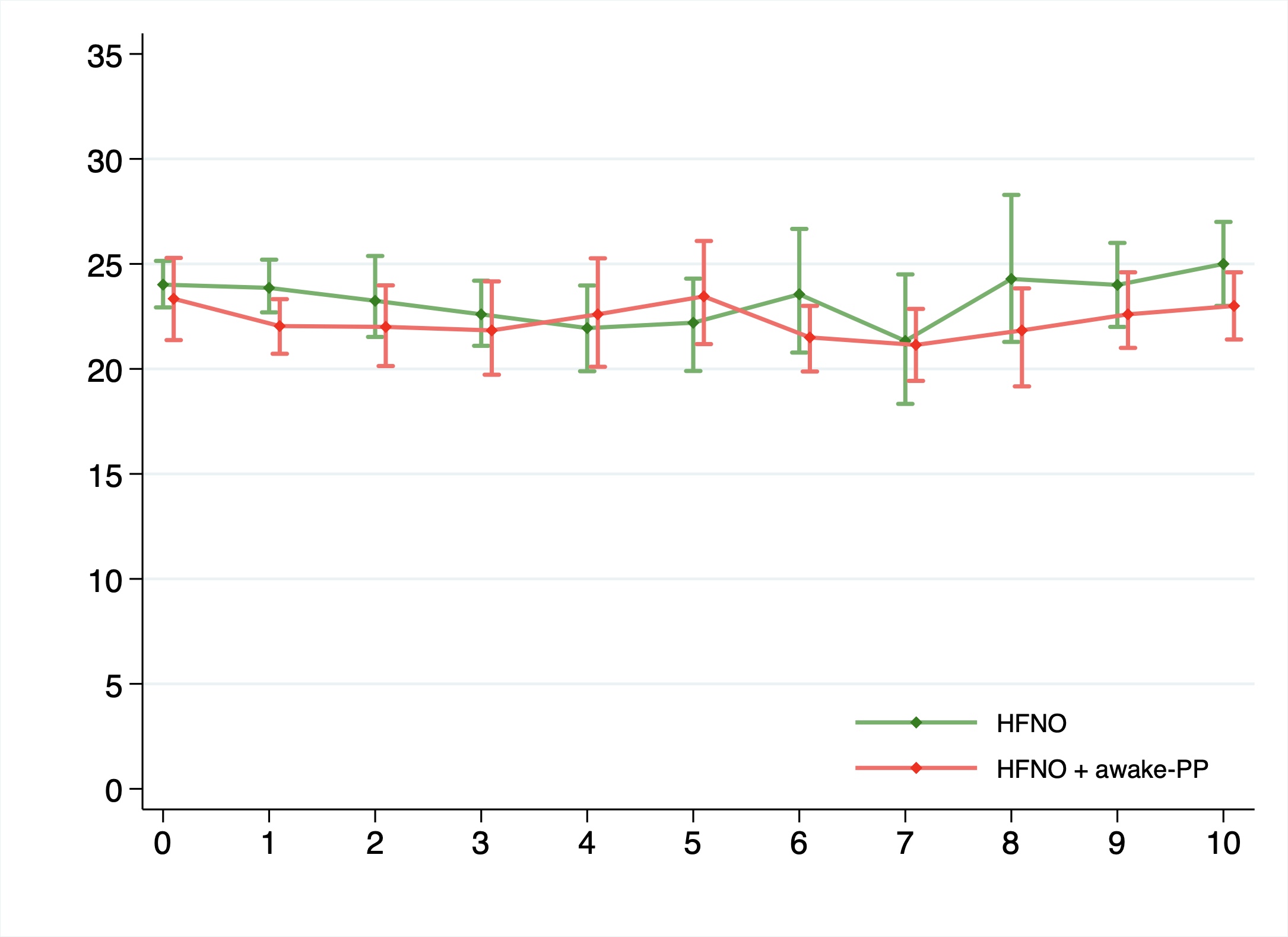


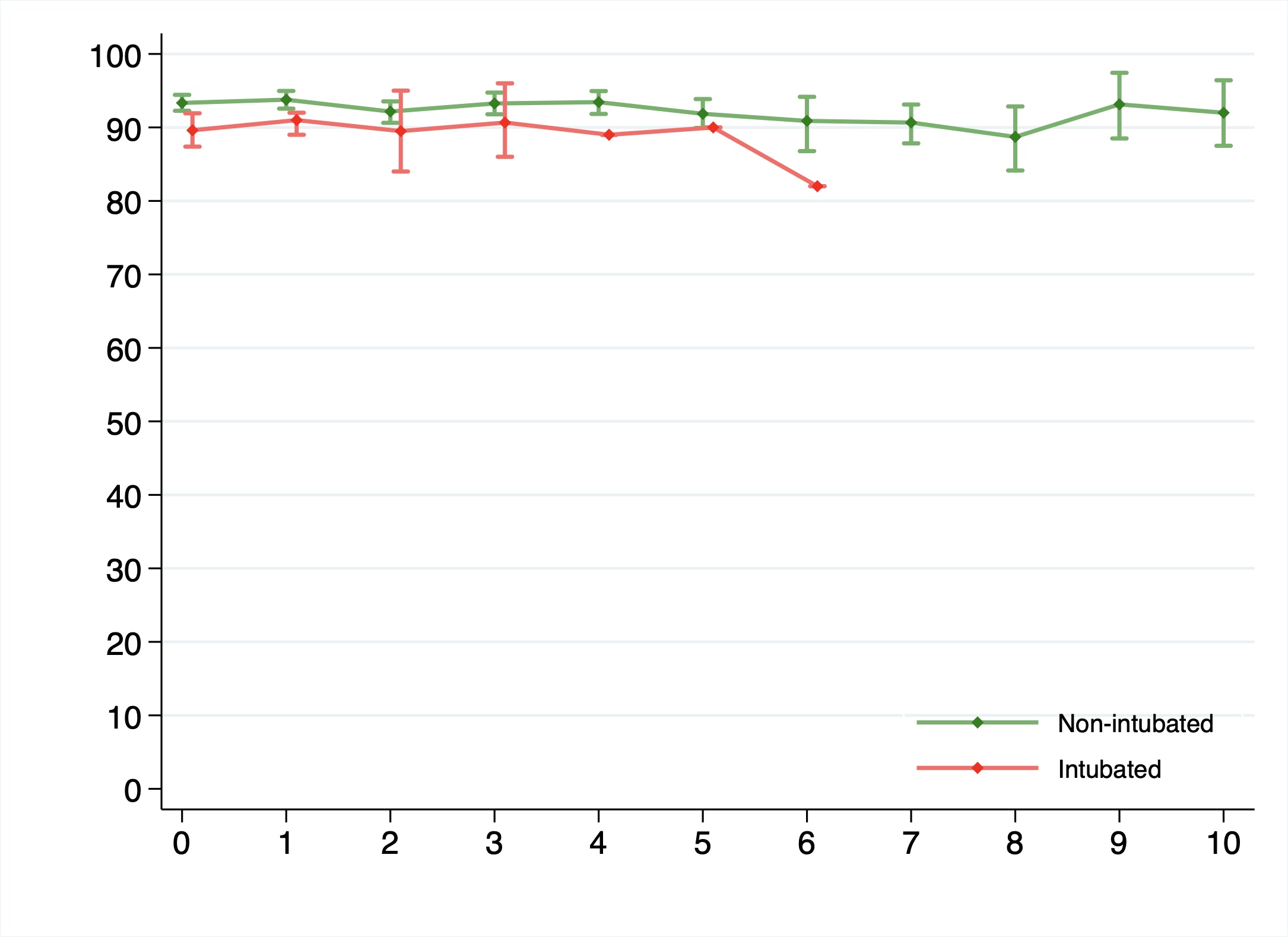

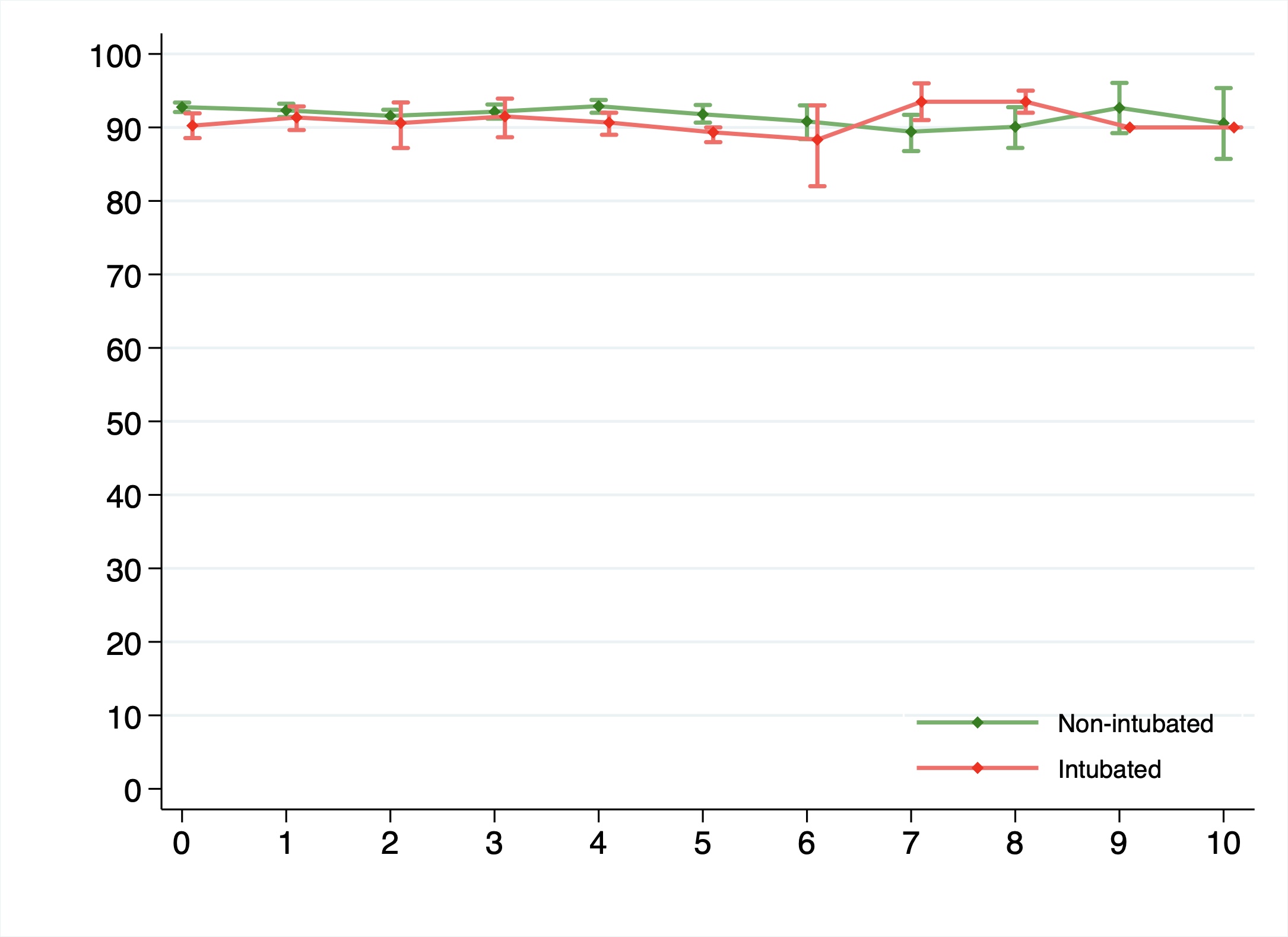


Figure 1. From top to bottom: HFNO vs HFNO+awake-PP, non-intubated vs intubated in patients with HFNO, non-intubated vs intubated in patients with HFNO+awake-PP. HFNO: High flow nasal oxygen therapy.

**Figure 2. Respiratory rate (breath per minute) over time in the adjusted population.**


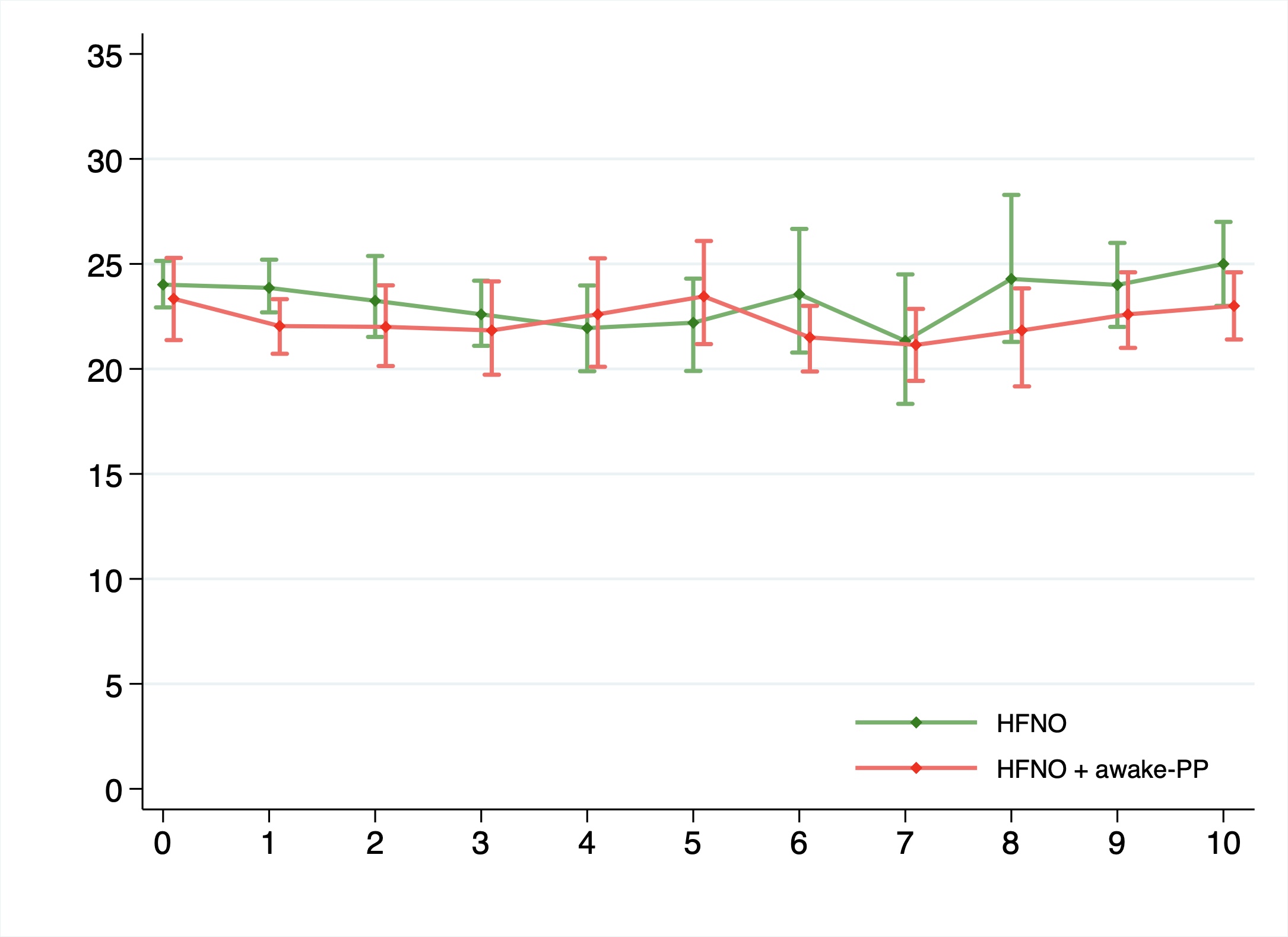


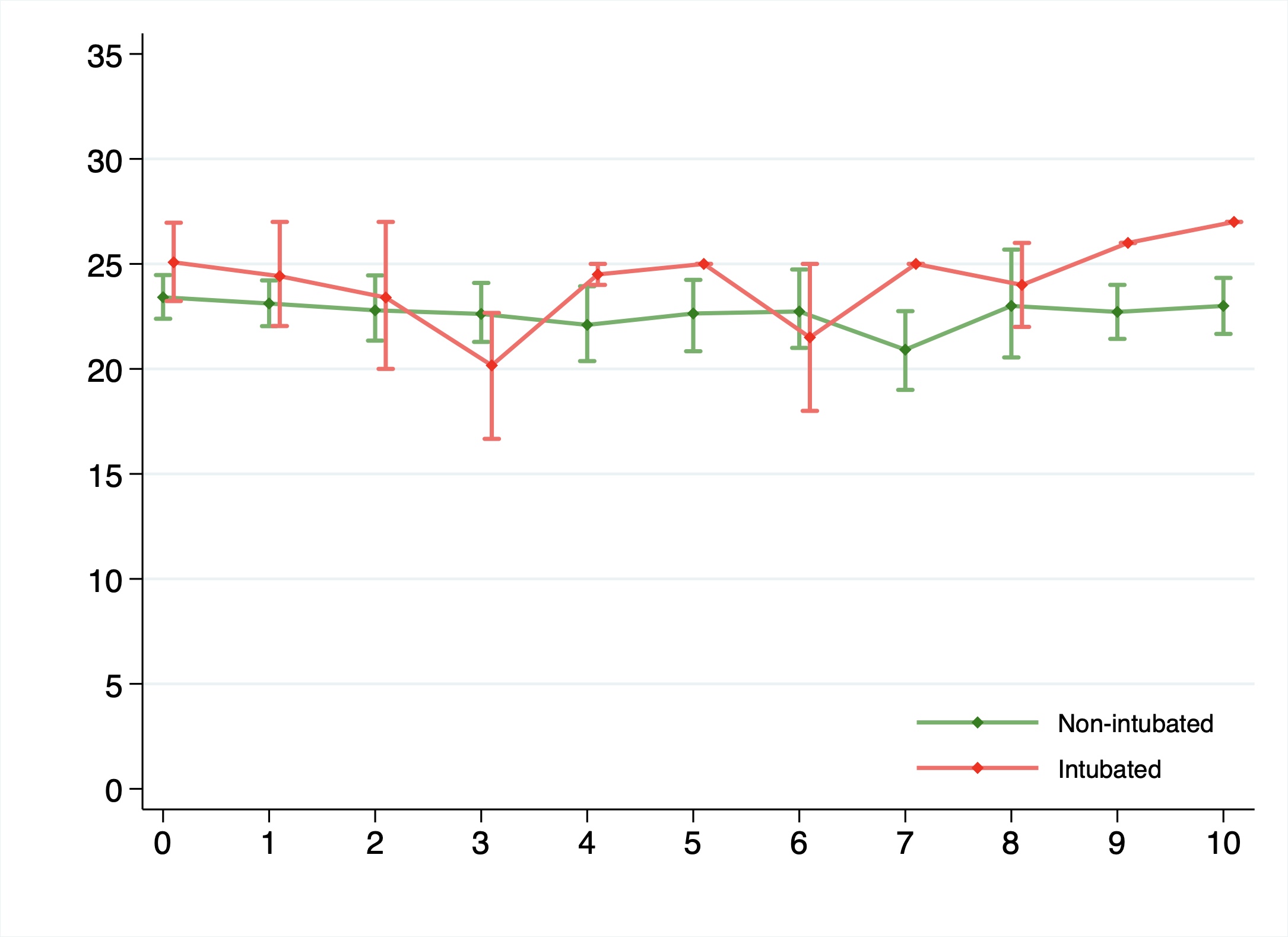


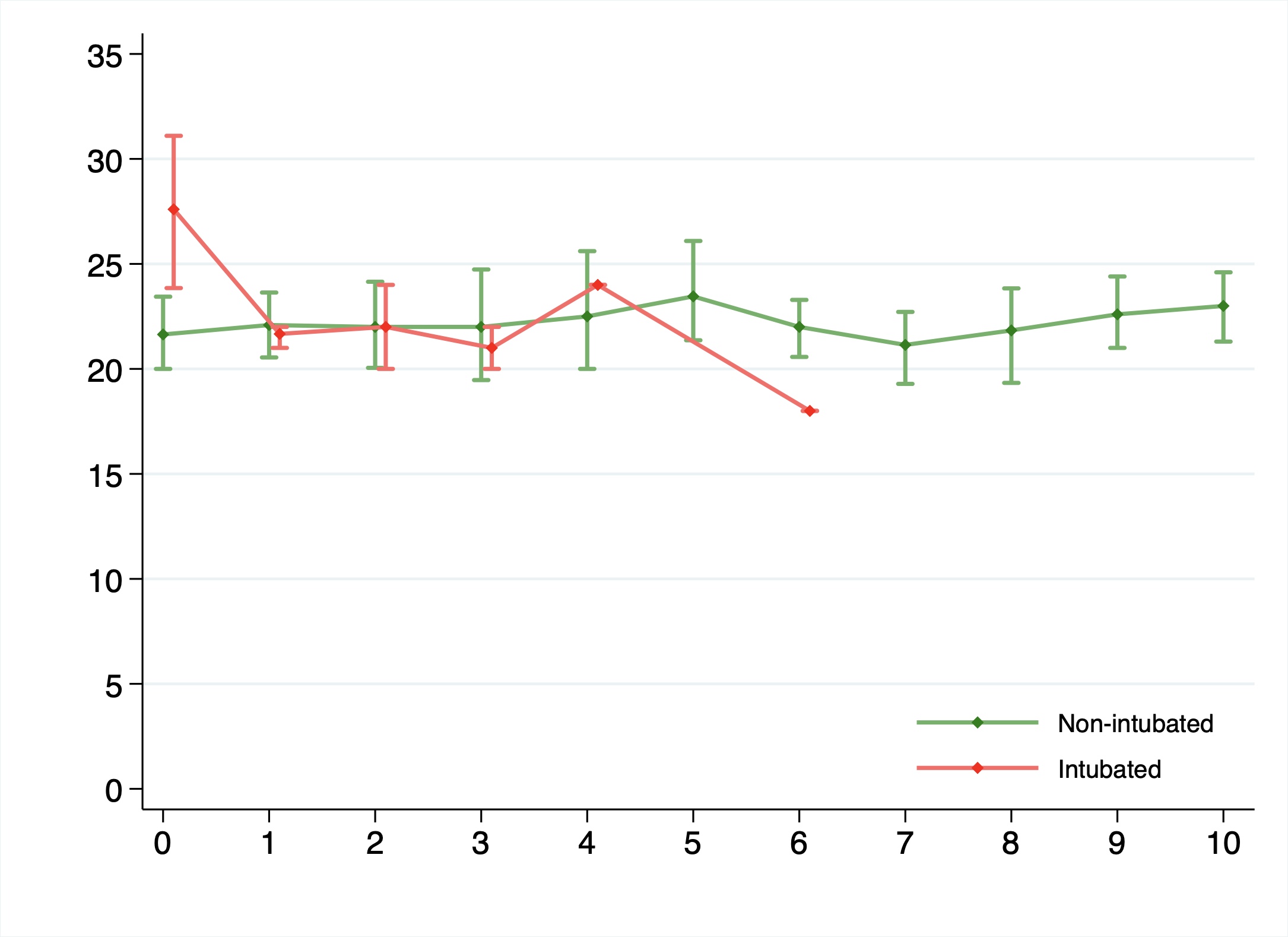


Figure 2. From to bottom: HFNO vs HFNO+awake-PP, non-intubated vs intubated in patients with HFNO, non-intubated vs intubated in patients with HFNO+awake-PP. HFNO: High flow nasal oxygen therapy.

**Figure 3. ROX Index [(SpO_2_/FiO_2_) / Respiratory rate] over time in the adjusted population.**


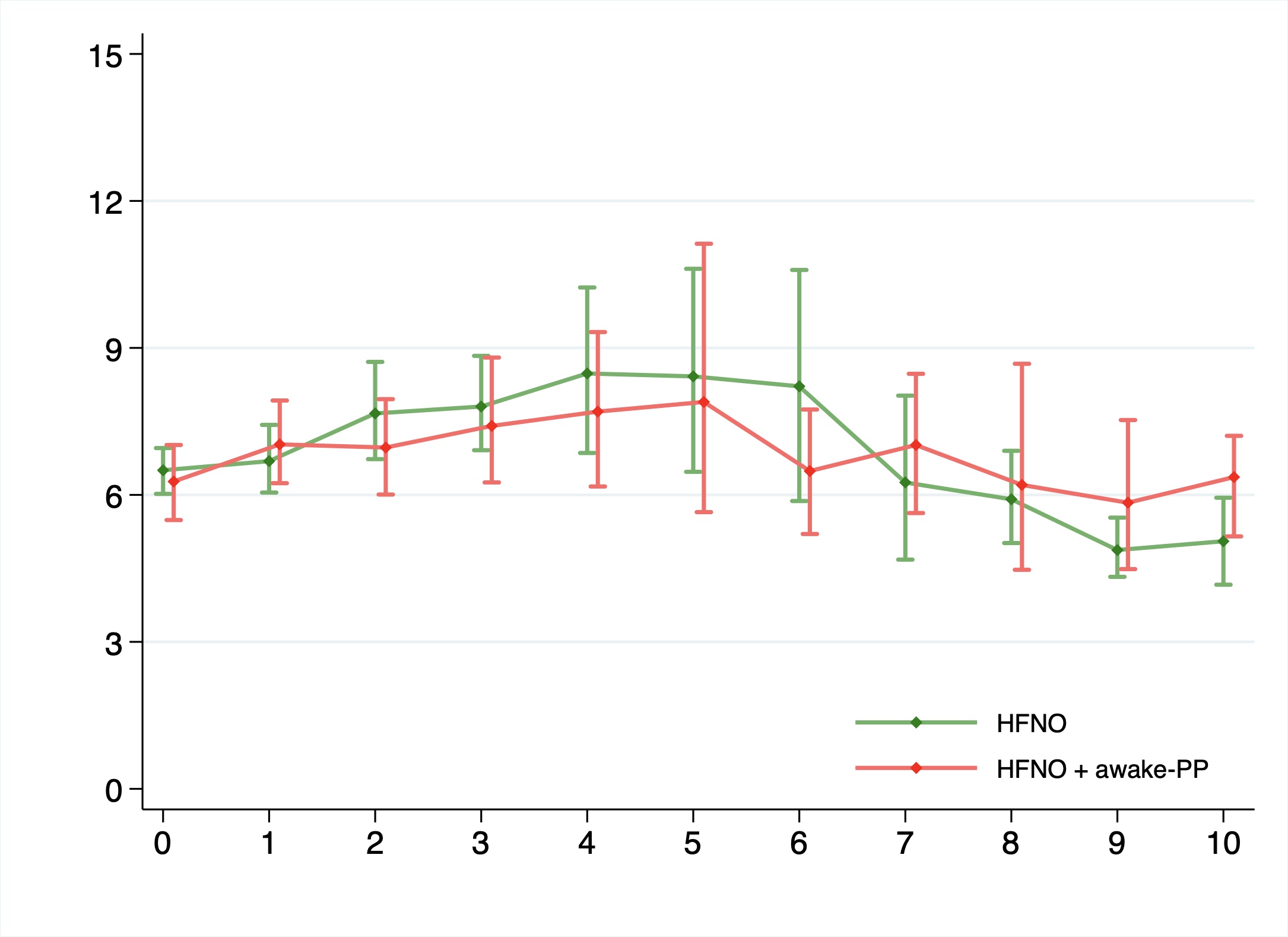


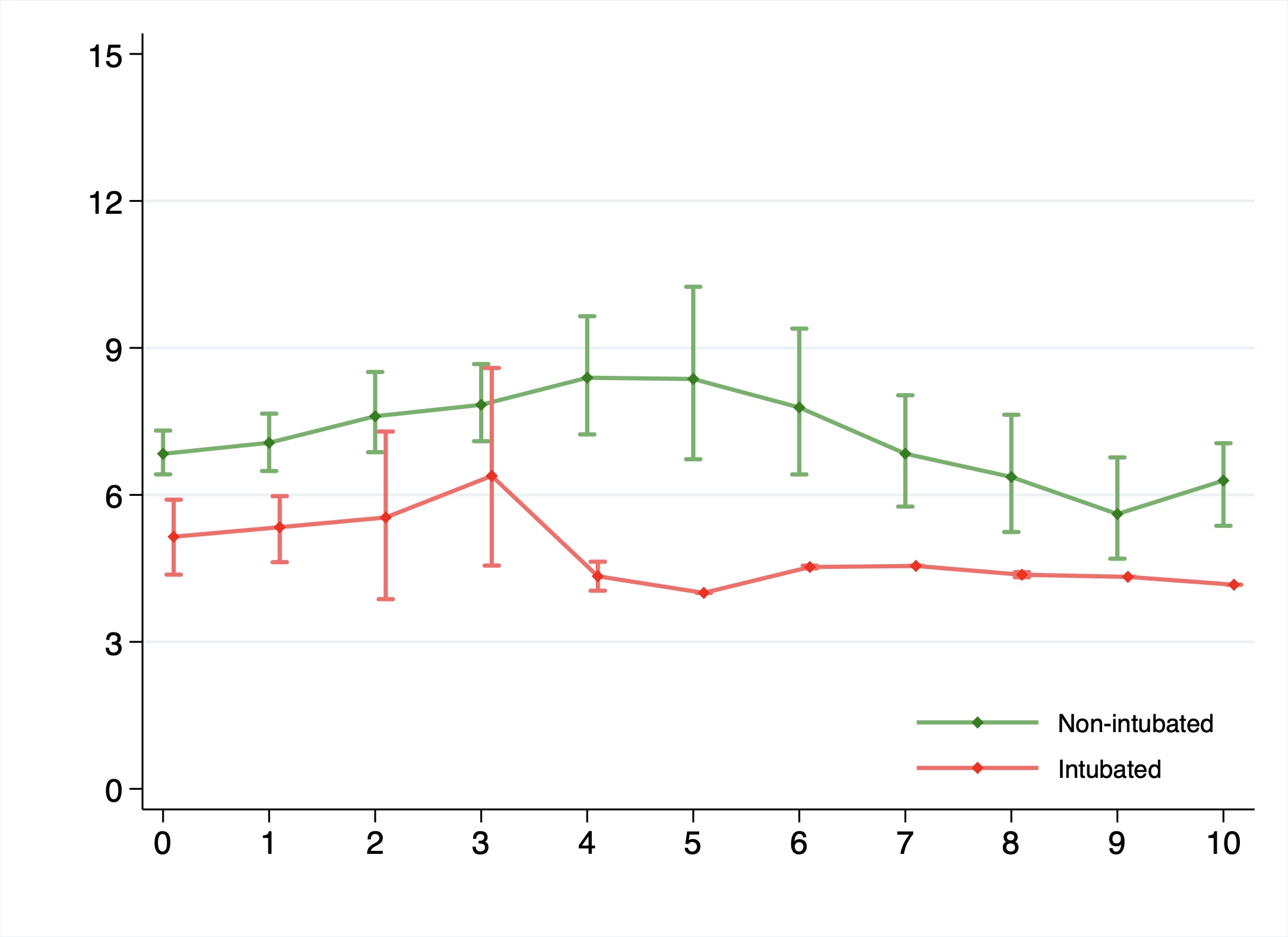


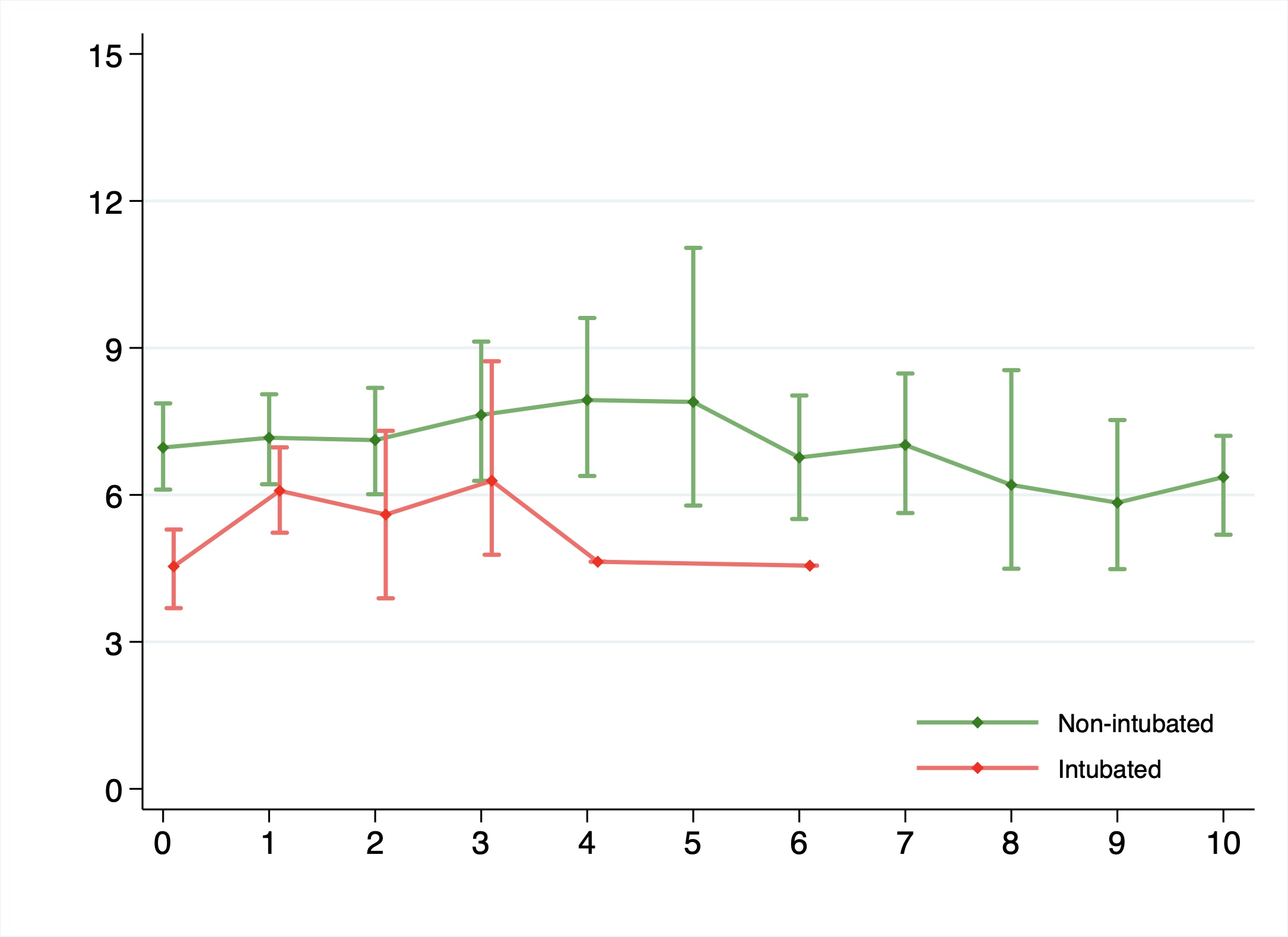


Figure 3. From to bottom: HFNO vs HFNO+awake-PP, non-intubated vs intubated in patients with HFNO, non-intubated vs intubated in patients with HFNO+awake-PP. HFNO: High flow nasal oxygen therapy.

**Figure 4. Probability of being intubated in patients stratified by PaO_2_/FiO_2_.**

Figure 4. Time to event curves using Kaplan-Meier with multivariate Cox regression. The probability of being intubated in the original samples. Top: Patients with baseline PaO_2_/FiO_2_ < 100; [HR 1.30 (95%CI: 0.59-2.88); p=0.51]. Bottom: Patients with baseline PaO_2_/FiO_2_ > 100; [HR 0.53 (95%CI: 0.25-1.50); p=0.10]. P-value for interaction=0.13.

**Figure 5. Probability of 28-day mortality in patients stratified by PaO_2_/FiO_2_.**

Figure 5. Time to event curves using Kaplan-Meier with multivariate Cox regression. The probability of 28-day mortality in the original samples. Top: Patients with baseline PaO_2_/FiO_2_ < 100; [HR 1.68 (95%CI: 0.33-8.66); p=0.53]. Bottom: Patients with baseline PaO_2_/FiO_2_ > 100; [HR 0.64 (95%CI: 0.12-3.32); p=0.59]. P-value for interaction=0.42.
